# Understanding the Influence of Electron Traps and Urbach States on the Kinetics of Ti^3+^ Persistent Luminescence in LaAlO_3_:Ti^3+^

**DOI:** 10.1021/acs.inorgchem.5c00390

**Published:** 2025-02-07

**Authors:** Wojciech M. Piotrowski, Justyna Zeler, Vasyl Kinzhybalo, Karolina Ledwa, Paulina Bukowska, Eugeniusz Zych, Lukasz Marciniak

**Affiliations:** † Institute of Low Temperature and Structure Research, Polish Academy of Sciences, Okólna 2, Wroclaw 50-422, Poland; ‡ Faculty of Chemistry, University of Wroclaw, 14.p F. Joliot-Curie Street, Wroclaw PL-50383, Poland

## Abstract

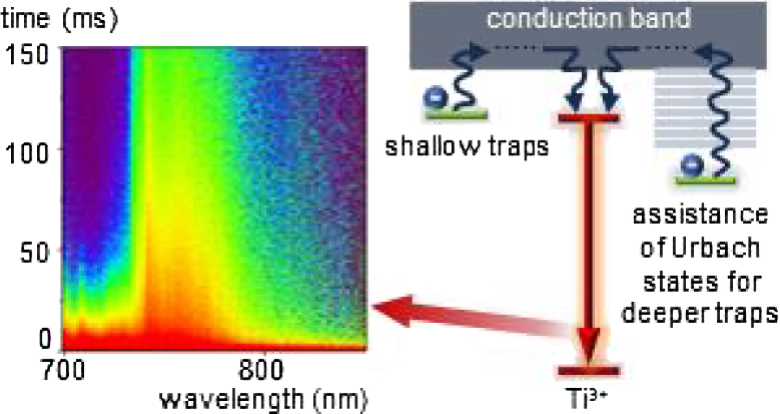

The luminescence kinetics of Ti^3+^ ions, resulting from the spin-allowed ^2^E → ^2^T_2_ electron transition, are generally expected to be fast, within the microsecond range. However, in this study, we observed average lifetimes of up to 30 ms in nanocrystalline LaAlO_3_:Ti^3+^ powders. Our detailed analysis of the spectroscopic and thermoluminescence properties of LaAlO_3_:Ti^3+^ suggests that this prolonged Ti^3+^ kinetics is associated with the presence of electron traps and the proximity of the Ti^3+^ excited state to the conduction band, which facilitates energy transfer between them. Furthermore, the observed shift in Urbach states with an increasing Ti^3+^ concentration correlates with the efficiency of energy transfer between deeper traps and Ti^3+^ ions. This study provides a comprehensive strategy for controlling the luminescence kinetics of Ti^3+^ ions through electron trap engineering, induced by dopant ion concentration, which can be applied in various fields including luminescence thermometry.

## Abstract 2

Ti^3+^ ions in cubic LaAlO_3_:Ti^3+^ are characterized by lifetimes of tens ms at room temperature. In this structure, the proximity of the excited ^2^E state to the conduction band enables electron transfer from the shallow electron traps to the ^2^E state, resulting in persistent luminescence. However, for Ti^3+^ concentration above 10%, the Urbach states assist in the improved emptying of deeper traps. This phenomenon leads to an even greater elongation of Ti^3+^ kinetics.

## Introduction

The lifetime of the excited state (τ) is one of the most important spectroscopic parameters used to identify and describe the optically active centers in phosphors.^1−9^ It is customarily described as the time after the excitation pulse within which the light intensity drops to 1*/e* of the initial value.^9^ Since the rate of excited level depopulation is sensitive to many different factors, luminescence kinetics analysis can provide much important information about changes in, e.g., temperature or pressure around the optically active center, or changes in the chemical environment of the phosphor, and thus serve as a remote indicator.^10−17^ However, in addition to the external stimuli mentioned above, the structural properties of the material in which the ion is stabilized also influence its kinetics. These include crystal symmetry, local site symmetry, crystal field strength, phonon energy, dopant concentration, occurrence and efficiency of energy transfers between ions, particle size, surface, and lattice defects in the structure, etc.^3,18−28^ In addition to quenching the luminescence, the latter can lead to the formation of electron or hole traps, which are one of the necessary conditions for the generation of persistent luminescence.^3,4,9,29−33^ In inorganic materials, traps most often arise from 1) interstitial ions or 2) vacancies, which are particularly expected in materials doped with ions of uncompensated ionic charge.^2,4,9,34,35^ However, intrinsic properties of some host materials, such as spinels and perovskites, may induce the formation of a third type of defect, namely antisites.^35−42^ This is when, in a structure with the general formula AB_2_X_4_ or ABX_3_, the A ions are located in the crystallographic positions of the B ions, or vice versa. An example of a structure in which all three types of defects mentioned can be observed is LaAlO_3_.^34,35,43−47^ Therefore, many reports on persistent luminescence in this host material can be found in the literature for LaAlO_3_ doped with multiple emitters, such as lanthanide ions: Pr^3+^,^48,49^ Er^3+^,^50^ Ho^3+^,^51^ Eu^3+^,^52^ and transition metals: Mn^4+^^44,45^ and Cr^3+^.^34,50,53^

In this study, the unique case of Ti^3+^ ions as dopants in a cubic LaAlO_3_ host material is presented. The *3d*^*1*^ electronic configuration of Ti^3+^ ions plays a crucial role in this case, leading to the observation of the unique spectroscopic properties of the LaAlO_3_:Ti^3+^ phosphor. First, the simplicity of the energy diagram of Ti^3+^ ions, consisting only of two energy levels, facilitates the interpretation of energy transfer mechanisms and reduces the possibility of intraionic nonradiative transitions. Moreover, the *3d*^*1*^ configuration provides a linear influence of the crystal field strength affecting Ti^3+^ ions on the spectroscopic parameters. Therefore, the concentration of Ti^3+^ ions, by changing the crystal field strength, affects the energy position of the excited state parabola and thus the position of the excitation and emission bands.

**Figure 1:**
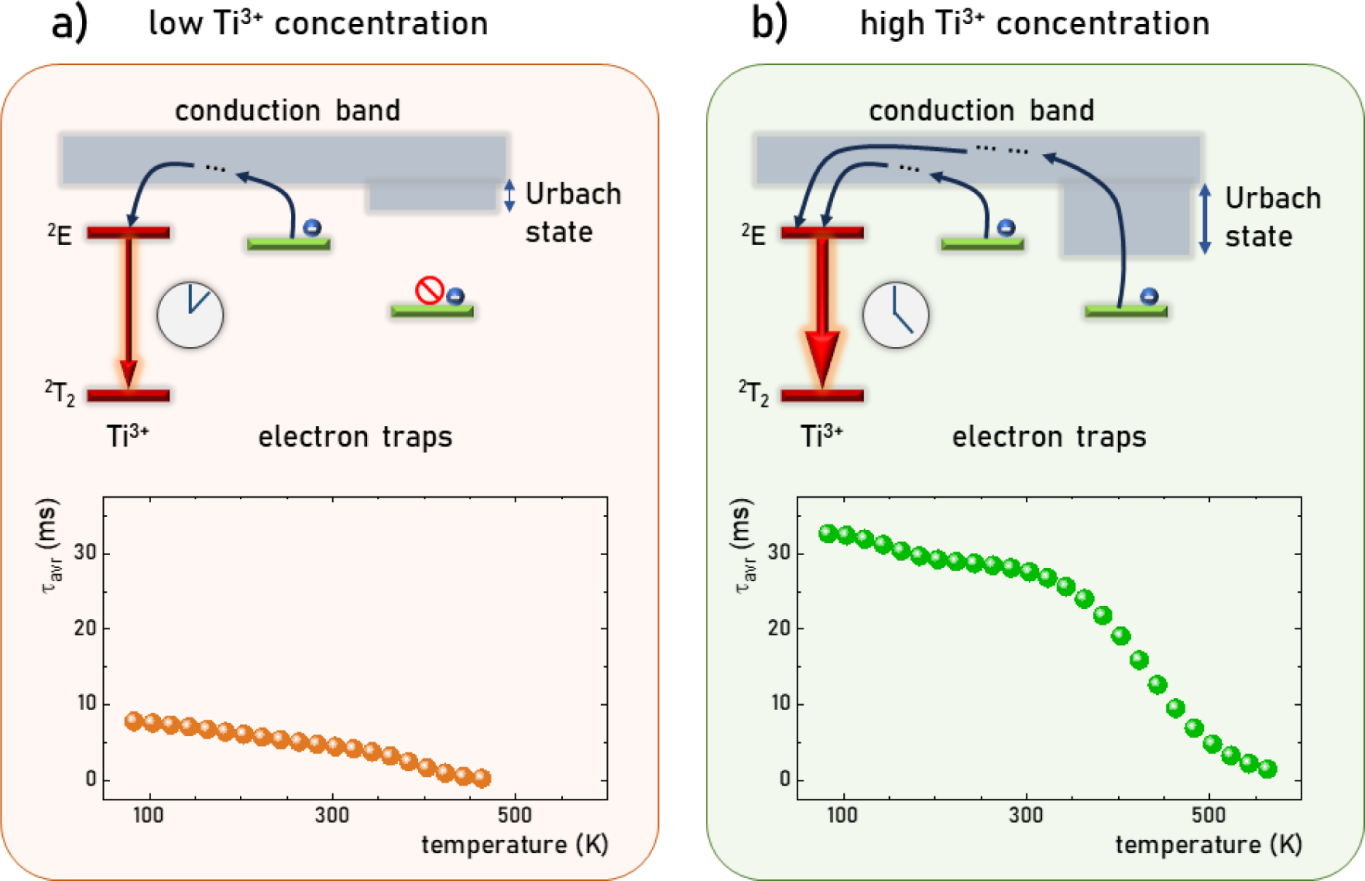
Visualization of the concept of the presented approach: comparison between electron transfer from shallow traps for low Ti^3+^ concentration (a) and electron transfer from deeper traps assisted by Urbach states for high Ti^3+^ concentration (b).

Furthermore, the previously reported preference for the occurrence of electron traps in the LaAlO_3_ structure may favor the observation of a Ti^3+^ persistent luminescence. As shown in this work, thermoluminescence measurements indicate a direct effect of electron traps on the elongation of the lifetime of the ^2^E excited state of Ti^3+^ ions in LaAlO_3_ by one/two orders of magnitude in respect to Ti^3+^ doped phosphors reported so far (Figure 1a).^54−61^ This mechanism requires the emptying of sufficiently shallow electron traps and electron transfer to the ^2^E excited state of Ti^3+^ via the conduction band. As was proven in this work, this mechanism is probable in LaAlO_3_, due to the close proximity of the ^2^E level to the conduction band.

The presence of Ti^3+^ also affects the occurrence of Urbach states associated with the deformation of the *d* states of La^3+^ ions. When Ti^3+^ ions gradually replace smaller Al^3+^ ions with increasing concentration, the Urbach states shift toward lower energies.^45,62−65^ Therefore, when the concentration of Ti^3+^ ions increases, the efficiency of filling and emptying of deeper traps may be enhanced by the assistance of Urbach states. This phenomenon may lead to an even greater elongation of the kinetics of Ti^3+^ ions when high dopant concentration is used in LaAlO_3_:Ti^3+^ with respect to low Ti^3+^ concentrations (Figure 1b). The hypothesis posed will be discussed in detail based on spectroscopic and thermoluminescence measurements. Understanding the mechanism of persistent luminescence generation in LaAlO_3_:Ti^3+^ allows the tuning of the luminescence kinetics of Ti^3+^ which is beneficial in many applications, i.e., lifetime-based luminescence thermometry.

## Experimental Section

### Synthesis

The powders of LaAl_(100-*x*)%_O_3_:*x*% Ti^3+^ (*x* = 0.1; 0.2; 0.5; 1; 2; 5, 10, 15, 20, 30, 50, 75), LaTiO_3_ were synthesized with a modified Pechini method.^66^ The following starting materials were used as reagents without further purification: Al(NO_3_)_3_·*x*H_2_O (*x* ≈ 99.999% purity, Alfa Aesar), Ti(OC_4_H_9_)_4_ (99+% purity, Alfa Aesar), 2,4-pentanedione (C_5_H_8_O_2_, 99% purity, Alfa Aesar), La_2_O_3_ (99.99% purity, Stanford Materials Corporation), citric acid (HOC(COOH)(CH_2_COOH)_2_, ≥ 99.5%, Sigma–Aldrich), polyethylene glycol (PEG-200, H(OCH_2_CH_2_)_n_OH, *n* = 200, Alfa Aesar), and HNO_3_ (65% solution, Avantor). The total amount of La^3+^ for the stoichiometric samples was fixed at 2 mmol. First, a stoichiometric amount of lanthanum oxide was dissolved in deionized water with the addition of 2 mL of HNO_3_ and then recrystallized three times to remove excess nitrogen. Subsequently, lanthanum nitrate was dissolved in deionized water, and 2–2*x*% mmol of aluminum nitrate and 24 mmol of anhydrous citric acid (with the molar ratio of citric acid to all metals set to 6:1) were added to the mixture. In a separate beaker, 2*x*% mmol of Ti(OC_4_H_9_)_4_ (amount determined with respect to the number of moles of Al^3+^ ions) was mixed with 2,4-pentanedione in a 1:1 molar ratio to stabilize the Ti(OC_4_H_9_)_4_ solution. The contents of the beaker were gently stirred until a transparent, yellowish solution was obtained, which was then combined with the stabilized nitrate solution. Finally, 24 mmol of PEG-200 was added to the mixture in a 1:1 molar ratio with respect to citric acid. The resulting solutions were then dried for 3 days at 363 K until resins were formed. The samples were then calcined in porcelain or corundum crucibles at different temperatures (heating rate of 5 K min^–1^) in air for 12 h. Finally, the obtained powders were ground in an agate mortar.

### Characterization

All synthesized materials were examined by powder X-ray diffraction (XRPD) measurements performed in Bragg–Brentano geometry on a PANalytical X’Pert diffractometer, using Ni-filtered Cu–Kα radiation (V = 40 kV, I = 30 mA). The Rietveld refinements of the XRPD patterns were performed using the X’Pert HighScore Plus version 2.2.4 software.

Transmission electron microscopy (TEM) images were taken using a Philips CM-20 SuperTwin microscope. The powders were ground in a mortar, dispersed in methanol with the aid of ultrasound, and deposited on standard copper grids covered with amorphous carbon. The studies were performed with a 160 keV parallel beam electron energy.

The quantitative composition of LaAlO_3_:Ti^3+^ phosphors was examined by using inductively coupled plasma optical emission spectroscopy (ICP-OES). ICP-OES analysis was carried out using an iCAP 7400 DUO ICP-OES Thermo Scientific spectrometer equipped with a high-performance solid-state charge injection device (CID) detector, the CID86, enabling the free choice of wavelengths in the 166–847 nm range. The experimental data were collected using Qtegra ISDS software. For the measurements, all powders were dissolved in 65% HNO_3_.

Thermoluminescence (TL) experiments were performed using the Lexsyg Research Fully Automated TL/OSL Reader from Freiberg Instruments GmbH. A VF-50J RTG X-ray lamp with a W-anode operated at 20–40 kV and 0.5 mA for 5–300 s or a UV–vis lamp (excitation at 365 nm) for 300 s was used as excitation/charging energy sources. The TL glow curves were recorded using a 9235QB type photomultiplier from ET Enterprises in the range of 303–723 K with a heating rate of 5 K s^–1^. All experiments were controlled by using LexStudio 2 software. The glow curves were deconvoluted into TL components using GlowFit software kindly supplied by Puchalska from the Institute of Physics PAS in Cracow, Poland.^67^

The excitation and emission spectra, luminescence decay profiles, and time-resolved emission/excitation spectroscopy (TRES) maps were recorded using the FLS1000 Fluorescence Spectrometer from Edinburgh Instruments with an R928 photomultiplier tube from Hamamatsu as a detector and a 450 W xenon lamp and 380 and 445 nm continuous/pulsed work laser diodes as excitation sources. The temperature of the sample was controlled using a Linkam THMS 600 heating–cooling stage (0.1 K temperature stability and 0.1 K set point resolution). The average lifetime of the excited states of Ti^3+^ was calculated with the biexponential function (eqs 1 and 2):
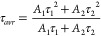
1where *τ*_1_ and *τ*_2_ are the decay parameters and *A*_1_ and *A*_2_ are the amplitudes of the biexponential function:
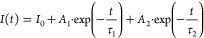
2

## Results and Discussion

### Structural and Morphological Characterization

**Figure 2:**
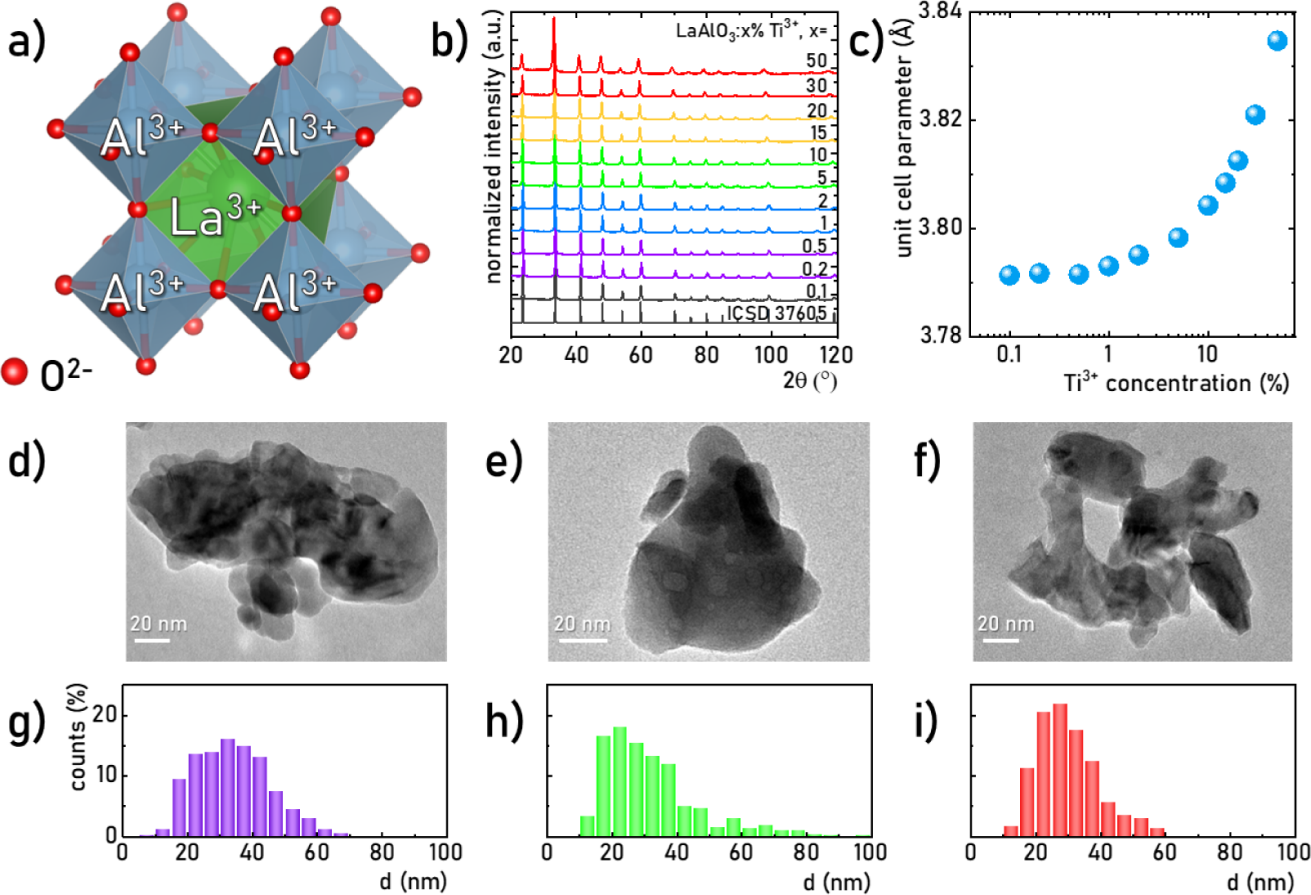
Visualization of the crystal structure of cubic LaAlO_3_ (a); the XRPD patterns of LaAlO_3_:Ti^3+^ doped with different Ti^3+^ concentration (b); the influence of Ti^3+^ concentration on the unit cell parameter of LaAlO_3_:Ti^3+^ (c); the representative TEM images (d–f) and particle size distributions (g–i) for LaAlO_3_ doped with 0.2% Ti^3+^ (d, g), 10% Ti^3+^ (e, h), and 50% Ti^3+^ (f, i).

Two crystal structures of LaAlO_3_ are described in the literature: rhombohedral with the  space group (No. 167) and cubic with the space group (No. 221).^68,69^ It is generally accepted that rhombohedral LaAlO_3_ is stable at room temperature and undergoes a phase transition to cubic above 800 ± 15 K^69^. The structure of both LaAlO_3_ forms consists of 6-fold coordinated Al^3+^ sites and 12-fold coordinated La^3+^ sites (Figure 2a). However, an important difference is that the LaAlO_3_ rhombohedral structure is characterized by a distortional rotation of the (AlO_6_)^9–^ octahedra around one of the triad axes with respect to the ideal cubic structure.^69,70^ Distinguishing between the two phases is particularly difficult since their XRD patterns are almost identical, differing only in the presence of additional weakly detectable reflections (e.g., at 2θ = ∼39.4 and ∼52.6°) and two overlapped peaks instead of one (e.g., ∼89.5, 94.2, and 98.9°) in the case of the rhombohedral phase (a comparison of ICSD 90533 and ICSD 37605 of rhombohedral and cubic LaAlO_3_, respectively, is shown in Figure S1). However, it should be emphasized that the previously indicated phase transition temperature is only true for single crystals, while for microcrystalline and nanocrystalline particles, this temperature is expected to decrease.^71−75^ A detailed study on this topic was carried out by Deren et al.,^68^ correlating particle size of nanometric LaAlO_3_ with the annealing temperature. The authors indicated that an annealing temperature of 1373 K or less allows one to obtain a material with a cubic structure, while exceeding 1473 K leads to the observation of rhombohedral forms at room temperature. Analysis of this issue continued by Tomaszewski^76^ led to the determination of a critical particle size *d*_cr_ = 60 nm, for which a cubic structure can be observed at room temperature (the phase transition will occur at 300 K).

Analysis of XRPD patterns of the LaAlO_3_:10% Ti^3+^ samples annealed at different temperatures revealed that the sample annealed at 973 K consists mainly of an amorphous phase (Figure S2). Residues of the amorphous phase are also evident for the sample annealed at 1073 K, for which observed narrow diffraction peaks are consistent with the reference pattern ICSD 37605 of the cubic LaAlO_3_ structure. The relatively broad diffraction reflections observed in the XRPD pattern suggest a small grain size of LaAlO_3_:Ti^3+^. However, the character of these reflections hinders the distinction between two separate reflections related to the rhombohedral phase. At higher annealing temperatures (between 1173 K and 1573 K), XRPD patterns of LaAlO_3_:Ti^3+^ do not indicate the presence of an amorphous phase, and no additional reflections are observed, suggesting well-crystallized pure LaAlO_3_. Also, in this temperature range, no features of the rhombohedral forms were distinguished, suggesting the presence of a cubic phase at room temperature. In reference to the spectroscopic parameters discussed later, an annealing temperature of 1273 K was selected as the optimum temperature for further studies. At this temperature, a series of LaAlO_3_:Ti^3+^ was prepared with the concentration of Ti^3+^ ions changed in the range of 0.1 to 100% with respect to Al^3+^ ions (Figure 2b, please check Figure S3 for 75% and 100% Ti^3+^). Preferential occupancy of Al^3+^ sites by Ti^3+^ ions is expected, due to the smaller difference in the Shannon effective ionic radii values (*R* = 67.0 pm, *R* = 53.5 pm, and *R* = 136.0 pm for Ti^3+^_VI_, Al^3+^_VI_, and La^3+^_XII_, respectively).^77^ It should be mentioned that the smallest difference between ionic radii was found for Ti^4+^_VI_, with *R* = 60.5 pm, for which the misfit factor *R*(Ti^4+^_VI_) – *R*(Al^3+^_VI_))/*R*(Al^3+^_VI_) = 11.6%. However, in this case, it is important to keep in mind the charge mismatch between the Al^3+^ and Ti^4+^ ions, which generates additional electron hole-type defects. This issue does not apply to Ti^3+^_VI_, which, despite a larger ionic radius than Al^3+^ ions and a misfit factor of 20.1%, is stabilized in the LaAlO_3_ structure, as confirmed by the spectroscopic studies presented in the following part of this manuscript. There is no additional crystalline phase present in LaAlO_3_:Ti^3+^ for concentrations of Ti^3+^ ions over a wide range of 0.1%–50%. For 75% Ti^3+^, additional reflections are consistent with the reference pattern ICSD 1950 of an orthorhombic La_2_Ti_2_O_7_ structure. The crystallization of La_2_Ti_2_O_7_ is even more evident for the stoichiometric sample of LaTiO_3_ (i.e., 100% Ti^3+^ substitution), and in this case, it is the dominant phase according to the XRPD pattern (Figure S3). Therefore, samples doped with 75% and 100% Ti^3+^ were not considered for further analysis. The Rietveld refinement provided additional information about the effect of the ratio of Ti^3+^ and Al^3+^ ions on the unit cell parameters (Table S1). The unit cell parameter *a* and, consequently, the volume of the unit cell increase with increasing Ti^3+^ concentration in the range between 0.1% and 50%, allowing the assumption that Ti^3+^ substitutes successively Al^3+^ ions (Figures 2c and S4). Furthermore, both the 6 equal Al/Ti^3+^- O^2–^ distances and 12 equal La^3+^-O^2–^ distances increase uniformly with increasing Ti^3+^ ion concentration from 0.1% to 50% (Figure S5). This demonstrates a uniform expansion of the unit cell without introducing additional distortion associated with the substitution of Al^3+^ ions by Ti^3+^.

ICP-OES measurements carried out for the Ti^3+^ concentration series of LaAlO_3_:Ti^3+^ indicate high compatibility between nominal and real Ti^3+^ ion concentrations (Table S2). The slight deviations (5%) of the total Al^3+^ and Ti^3+^ contents exceeding 100% with respect to La^3+^ are probably due to the hygroscopic properties of La_2_O_3_, which was the precursor for the La^3+^ ions.^78,79^ TEM studies of representative LaAlO_3_ with 0.2%, 10%, and 50% of Ti^3+^ ions annealed at 1273 K indicate well-crystallized and mainly agglomerated grains (Figures 2d-f and S6–S8). It can be seen that high Ti^3+^ concentrations lead to a decrease in the degree of agglomeration of grains. The histograms of Feret diameter indicate that the volume of samples with 0.2%, 10%, and 50% of Ti^3+^ ions predominates with grains smaller than 40 nm (Figures 2g–i). A slight tendency toward smaller grain sizes with increasing Ti^3+^ ion concentration is observed, but this is of negligible significance with respect to the observed grain size distribution. The determined average grain sizes are equal to 34 ± 11, 32 ± 14, and 30 ± 10 nm for 0.2%, 10%, and 50% of Ti^3+^ ions, respectively. An extrapolation of an approach presented in ref.^76^ leads to the conclusion that for grain sizes below 40 nm, a phase transition would be expected at temperatures below 150 K. However, based on spectroscopic studies in the temperature range above 83K presented below, no indication of a phase transition was observed.

### Luminescent Properties Characterization

**Figure 3:**
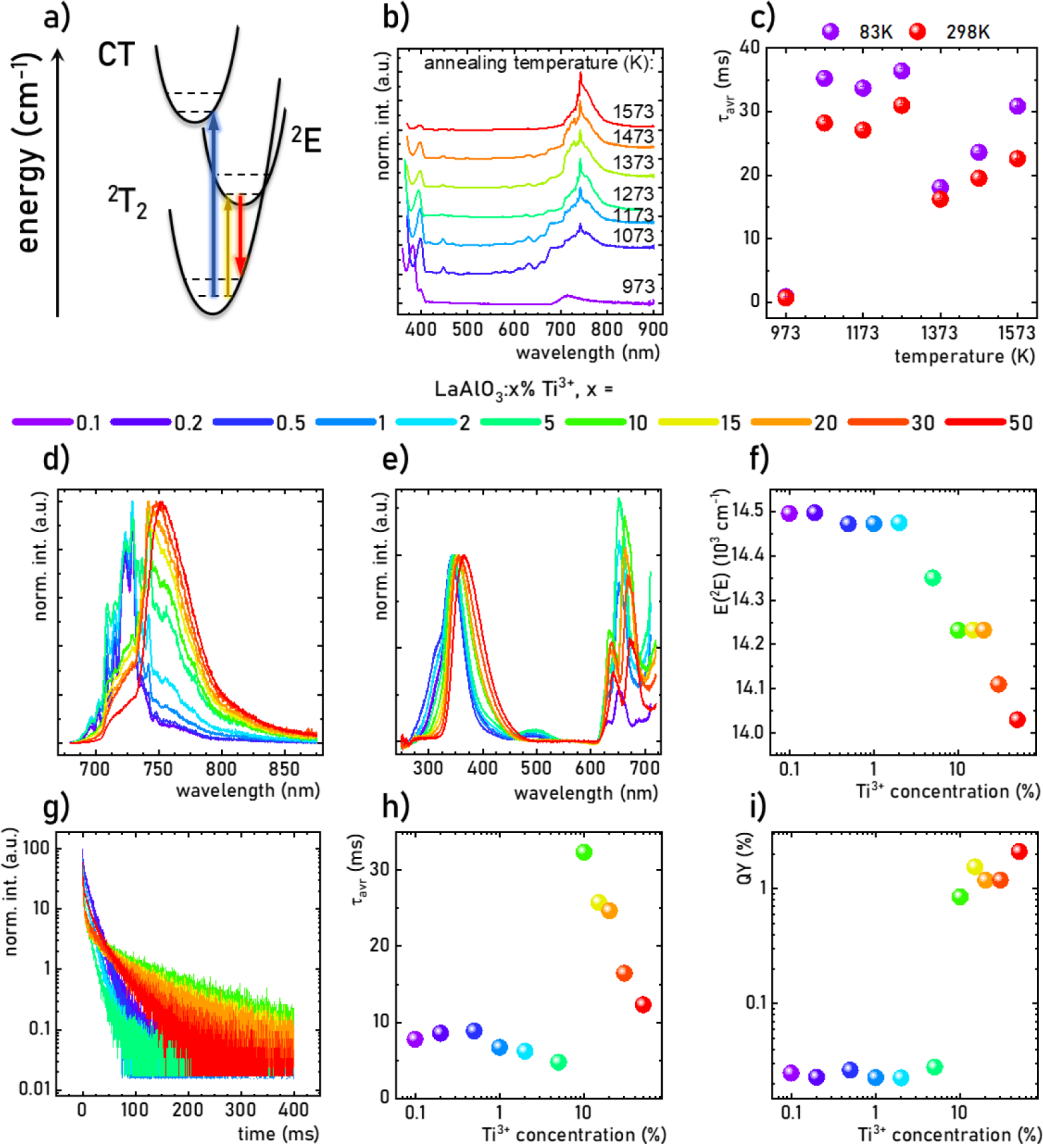
Simplified configurational coordination diagram of Ti^3+^ ions (a); comparison of the emission spectra of LaAlO_3_:10% Ti^3+^ annealed at different temperatures (*T* = 83 K, λ_exc_ = 351 nm) (b); the influence of the annealing temperature on the τ_avr_ of ^2^E(Ti^3+^) measured at 83 K and 298 K (λ_exc_ = 351 nm, λ_em_ = 742 nm) (c); the influence of Ti^3+^ dopant concentration on emission spectra of LaAlO_3_:Ti^3+^ (*T* = 83 K) (d), excitation spectra (*T* = 83 K) (e), energy position of ^2^E state of Ti^3+^ ions (f), luminescence decay profiles (g), τ_avr_ of ^2^E(Ti^3+^) (h), and luminescence quantum yield (i) of LaAlO_3_:Ti^3+^ samples.

Ti^3+^ ions are characterized by a *3d*^*1*^ electronic configuration. Their configurational coordinate diagram at the octahedral site consists of only two energy levels: the ground ^2^T_2_ and the excited ^2^E (Figure 3a).^80,81^ Therefore, excitation of electrons in the Ti^3+^ ions leads to the population of the ^2^E level (yellow arrow in Figure 3a), which is followed by nonradiative relaxation to the bottom of the ^2^E parabola. Subsequently, radiative depopulation leads to the generation of red emission (red arrow). However, if a sufficiently higher energy is applied, the O^2–^ → Ti^4+^ charge transfer transition (blue arrow) may also lead to the population of the ^2^E state of the Ti^3+^ ions. To investigate the effect of annealing temperature on the spectroscopic properties of LaAlO_3_:10% Ti^3+^, their low-temperature (83 K) emission spectra were compared (Figure 3b). All spectra were found to consist of two bands: one around 390 nm related to the Ti^4+^ → O^2–^ charge transfer transition and another broad band with the maximum at 742 nm related to the ^2^E → ^2^T_2_ electronic transition. The ratio of their intensities clearly increases with an increasing annealing temperature, which may indicate that a high annealing temperature may increase the Ti^3+^ content (Figure S9). This hypothesis can be confirmed by comparing the excitation spectra measured at *λ*_em_ = 742 nm (Figure S10). The broad band around 670 nm related to ^2^T_2_ → ^2^E transition is clearly more intense with respect to the O^2–^ → Ti^4+^ charge transfer band for the sample annealed at 973 K compared to higher temperatures. Luminescence decay profiles for the ^2^E(Ti^3+^) state are also significantly shorter for the sample annealed at 973 K compared to other samples, probably related to the high probability of nonradiative depopulation of the excited state associated with the low crystallinity of the sample (Figure S11). To perform a comparative analysis of the influence of annealing temperature on the kinetics of Ti^3+^ luminescence, the calculation of average lifetimes according to eqs 1 and 2 was performed. For the sample annealed at 973 K, *τ*_avr_ was 0.87 and 0.68 ms at 83 and 298 K, respectively (Figure 3c). However, an increase in the annealing temperature results in more than a 20-fold elongation of the kinetics of the Ti^3+^ state. The highest values, i.e., *τ*_avr_ = 32.36 ms at 83 K and 30.98 ms at 298 K, were obtained for LaAlO_3_:10% Ti^3+^ annealed at 1273 K, and therefore, further studies focus on the sample annealed at this temperature.

The influence of the Ti^3+^ concentration on emission spectra is depicted in Figure 3d. The spectrum of 0.1% Ti^3+^ consists of a broad band with several local maxima, the most intense of which is at 729 nm (∼13717 cm^–1^). A similar emission band shape remains for Ti^3+^ concentrations between 0.1% and 2%. However, above this value, the emission maximum shifts with increasing Ti^3+^ ion concentration toward longer wavelengths, reaching about 752.5 nm (13277 cm^–1^) for 50% Ti^3+^ (Figure S12). The observed red shift of the emission band results from the change in crystal field strength with the gradual replacement of smaller Al^3+^ ions by larger Ti^3+^ ions in the LaAlO_3_ structure. This effect is expected since, according to the Tanabe–Sugano diagram for TM ions with a *3d*^*1*^ electronic configuration, a linear dependence of ^2^E level energy on crystal field strength can be found. Above 2% Ti^3+^, an increasing contribution of the broad band is clearly found in the shape of the ^2^E → ^2^T_2_ band, which gradually starts to dominate. For LaAlO_3_:30% and LaAlO_3_:50% Ti^3+^, this broad band is the only component in the emission spectrum. A similar effect of Ti^3+^ ion concentration was observed in the case of the excitation spectra of LaAlO_3_:Ti^3+^ (Figure 3e). Both the bands associated with the ^2^T_2_ → ^2^E transition and the CT band shift progressively from 653 nm (15300 cm^–1^) to 676 nm (14780 cm^–1^) and from 343 (29150 cm^–1^) to 368 nm (27150 cm^–1^), respectively, as the concentration of Ti^3+^ increases (Figure S13). A small difference in the spectral shift rate between the ^2^T_2_ → ^2^E band in the excitation spectrum and the ^2^E → ^2^T_2_ band in the emission spectrum results in only a small change in the Stokes shift between their maxima from 1510 to 1560 cm^–1^ when the dopant concentration was changed from 0.1% Ti^3+^ to 50% Ti^3+^. The Stokes shift value in LaAlO_3_:Ti^3+^ is smaller than for Ti^3+^ doped Al_2_O_3_, YAlO_3_, and Y_3_Al_5_O_12_^54,55^ but is similar to that noted for BeAl_2_O_4_:Ti^3+57^ (Table S3). The energy of the ^2^E parabola, determined as the arithmetic mean of the position of ^2^T_2_ → ^2^E and ^2^E → ^2^T_2_ bands, decreases from 14495 to 14030 cm^–1^ as an increasing function of Ti^3+^ concentration (Figure 3f). Additionally, the monotonic decrease in the intensity of the CT band relative to the ^2^E → ^2^T_2_ band may indicate an increase in the efficiency of pumping the ^2^E level of Ti^3+^ ions with an increasing Ti^3+^ concentration (Figure S14). Moreover, a similar effect of Ti^3+^ concentration on CT emission and CT excitation band maxima was observed (Figure S15a). The luminescence decay profiles of CT emission are almost independent of Ti^3+^ ion concentration in LaAlO_3_:Ti^3+^ with the *τ*_avr_ in the range of 1.6–2.0 ms (Figure S15b,c).

A deeper analysis of the spectroscopic properties of Ti^3+^ ions led to measurements of the luminescence decay profiles of the ^2^E(Ti^3+^) excited state (Figure 3g). An elongation of one component is clearly observed for a concentration of 10% Ti^3+^ with respect to lower concentrations, which then gradually decreases as the concentration increases from 10% to 50%. A quantitative comparison of *τ*_avr_ indicated that *τ*_avr_ = 7.78 ms at 83 K was determined for LaAlO_3_:0.1% Ti^3+^ (Figure 2h), which is one or even 2 orders of magnitude longer than most *τ*_avr_ for Ti^3+^-doped phosphors reported in the literature to date (the comparison was presented in Table S4).^54−61^ This indicates the presence of additional processes that affect the kinetics of Ti^3+^ in LaAlO_3_. Thereafter, *τ*_avr_ increases slightly for 0.5% Ti^3+^ and gradually decreases to 4.77 ms for 5% Ti^3+^. However, an almost 7-fold prolongation of the *τ*_avr_ value to 32.36 ms was found for 10% Ti^3+^. This suggests a change in the nature of the kinetics of the ^2^E luminescence above this value of the dopant concentration. A further increase in Ti^3+^ concentration leads to sublinear shortening of *τ*_avr_ to 12.31 ms for LaAlO_3_:50% Ti^3+^. An abrupt change was also observed in the Ti^3+^ luminescence intensity between LaAlO_3_ doped with 5% and 10% Ti^3+^ ions. A quantum yield (QY) of 0.023–0.028% was achieved for LaAlO_3_ doped with 0.1–5% Ti^3+^ ions (Figure 3i). However, when 10% Ti^3+^ was added, the QY value grew to 0.85% and remained above this level for higher Ti^3+^ concentrations, reaching QY = 2.10% for 50% Ti^3+^. This is further confirmation of the significant change in spectroscopic properties of LaAlO_3_:Ti^3+^ that occurs between 5% and 10% Ti^3+^ concentration.

Host materials containing Al^3+^ often face nonintentional Cr^3+^ impurities due to the presence of Cr^3+^ in Al^3+^ precursors. Many Al^3+^-based host materials (including LaAlO_3_) contain trivalent octahedral sites that are favorable for stabilizing optically active Cr^3+^ ions. Since the spectral range of Cr^3+^ emission overlaps with that of Ti^3+^ and the lifetime of the ^2^E level of Cr^3+^ ions is significantly longer than expected for ^2^E of Ti^3+^ ions, it is crucial to verify whether Cr^3+^ ions are present in the material and whether they affect the spectroscopic properties of Ti^3+^ ions. For this purpose, emission spectra upon *λ*_exc_ = 445 nm, which is commonly applied for Cr^3+^ ion excitation, were performed. Comparison of the emission spectra of LaAlO_3_:Ti^3+^ obtained upon *λ*_exc_ = 445 nm and *λ*_exc_ = 380 nm excitations reveals that for 0.1% Ti^3+^, a narrow emission band associated with ^2^E → ^4^A_2_ of Cr^3+^ ions with a maximum at 734 nm is clearly detectable (Figure S16). This band does not spectrally overlap with the emission of Ti^3+^ ions (maximum at 729 nm). Moreover, upon 380 nm excitation, the emission intensity of the Cr^3+^ ions is negligible. The ^2^E → ^4^T_2_ band remains dominant upon *λ*_exc_ = 445 nm up to 15%Ti^3+^. Above 5% Ti^3+^, the intensity of the broad ^2^E → ^2^T_2_ band of Ti^3+^ ions gradually increases with respect to the narrow ^2^E → ^4^A_2_ band of Cr^3+^ ions. The position of the broad ^2^E → ^2^T_2_ band is consistent with that observed at 380 nm excitation, which is particularly well emphasized by the almost identical shape of the emission band at 380 and 445 nm excitation for LaAlO_3_:50% Ti^3+^. However, even more relevant is the comparison of the kinetics of the ^2^E(Ti^3+^) state with the ^2^E(Cr^3+^) one. It was found that Cr^3+^ decay profiles are noticeably shorter than their Ti^3+^ counterparts for all of the analyzed Ti^3+^ concentrations (Figure S17). The similar values of τ_avr_ of the ^2^E state of Cr^3+^ ions for samples doped with 0.1% to 50% Ti^3+^ ions may suggest that the impurity of Cr^3+^ ions originates from the Al^3+^ precursor (aluminum nitrate) and does not depend on the amount of Ti^3+^ ions introduced. The negligible effect of Ti^3+^ concentration on the shortening of the τ_avr_ of the ^2^E(Cr^3+^) with respect to τ_avr_ of ^2^E(Ti^3+^) suggests the lack of Cr^3+^ → Ti^3+^ energy transfer and thus no participation of Cr^3+^ in the population of the ^2^E state of the Ti^3+^ state (Figure S17c). This unequivocally confirms that the presence of Cr^3+^ ions in lanthanum aluminate has a negligible effect on the spectroscopic properties of Ti^3+^ ions and allows further analysis to ignore them from consideration.

**Figure 4:**
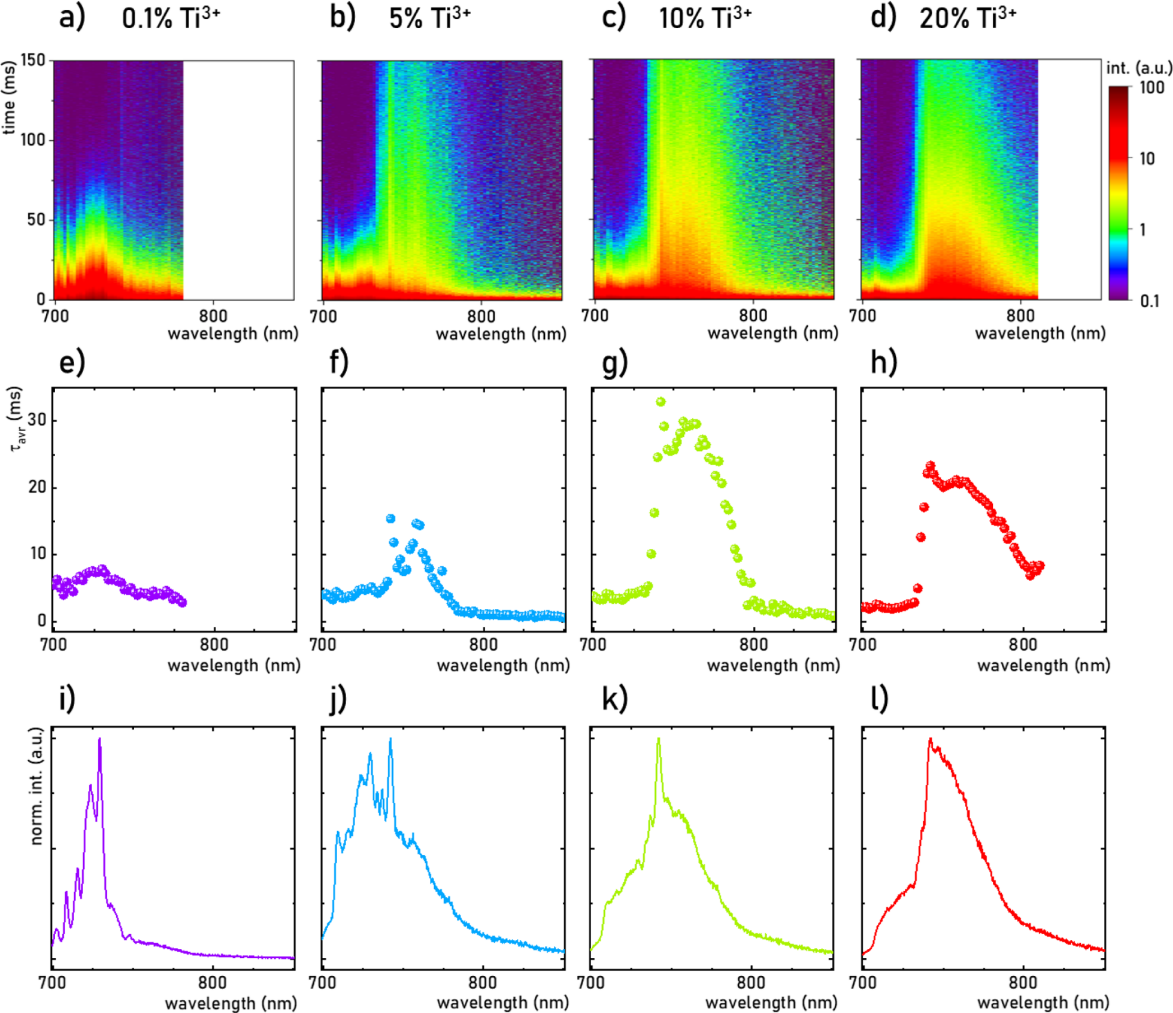
Comparison of the TRES map (*T* = 83 K) (a–d), corresponding τ_avr_ of ^2^E(Ti^3+^) as a function of λ_em_ (e–h) and emission spectra (i–l) carried out at 83 K for representative LaAlO_3_ doped with 0.1% Ti^3+^ (a, e, i, λ_exc_ = 346 nm), 5% Ti^3+^ (b, f, j, λ_exc_ = 351 nm), 10% Ti^3+^ (c, g, k, λ_exc_ = 351 nm), and 20% Ti^3+^ (d, h, l, λ_exc_ = 355 nm).

A more accurate depiction of the luminescence of Ti^3+^ ions in the LaAlO_3_ host is provided by TRES performed for several representative Ti^3+^ concentrations (Figures 4a–d). It can be clearly observed that the luminescence decay profiles performed for 0.1% Ti^3+^ are shorter than the counterparts obtained for 5% Ti^3+^ and 10% Ti^3+^. This is consistent with the τ_avr_ value of ^2^E(Ti^3+^) obtained (Figures 4e–h). In all materials, no temporal change in the shapes of the emission band was observed, which may indicate the presence of only one optical center. The spectral shift of the longest decay profile toward longer wavelengths with increasing Ti^3+^ concentration is also consistent with the corresponding emission spectra (Figures 4i–l). In contrast, for 20% Ti^3+^, the emission band shape remains broader, with no additional narrow bands, but with lower τ_avr_ values with respect to 10% Ti^3+^. A rarely observed dependence of τ_avr_ on λ_exc_ demonstrated in the form of TRES maps for a representative LaAlO_3_:10% Ti^3+^ suggests an influence of λ_exc_ on additional nonradiative processes preceding depopulation of the ^2^E(Ti^3+^) level, such as electron trap occupancy (Figure S18).

### Thermoluminescence Characterization

Such unexpectedly long decay times in LaAlO_3_:Ti^3+^ suggest their relation to the presence of trap states interfering with excited electrons in the structure. Therefore, thermoluminescence measurements were conducted using 365 nm irradiation and X-rays as charging sources to reveal the trapping sites capable of interacting with the excited electrons. To gain a complete understanding of electron trap energy levels in the material, several TL techniques were performed on selected concentrations of 0.2% Ti^3+^ (Figure S19 a–d), 10% Ti^3+^ (Figure S19 e–h), and 50% Ti^3+^ (Figure S19 i–l) in the LaAlO_3_:Ti^3+^ phosphors. The most intense TL was shown by the diluted material (0.2% of Ti^3+^). The complex structure of the TL curves evidences the presence of several traps of different depths in each phosphor. The TL intensity of all three phosphors increases with the longer irradiation time (Figure S19a, e, and i). Importantly, the shape of the glow curves remains unchanged regardless of the dose; the peak positions are firmly at the same temperatures, proving the first-order kinetics of the TL process in all materials.^82,83^ Even if the shape of the glow curves changes with Ti^3+^ content, it is easy to note that in all compositions, very similar components are observed, indicating the presence of similar traps. Based on the obtained results, three types of traps can be distinguished, and the respective TL peak maxima appear at around 340, 405, and 490 K. Whether the different TL components represent independent, individual traps or come from traps showing energy distribution will be analyzed below.^84^

**Figure 5:**
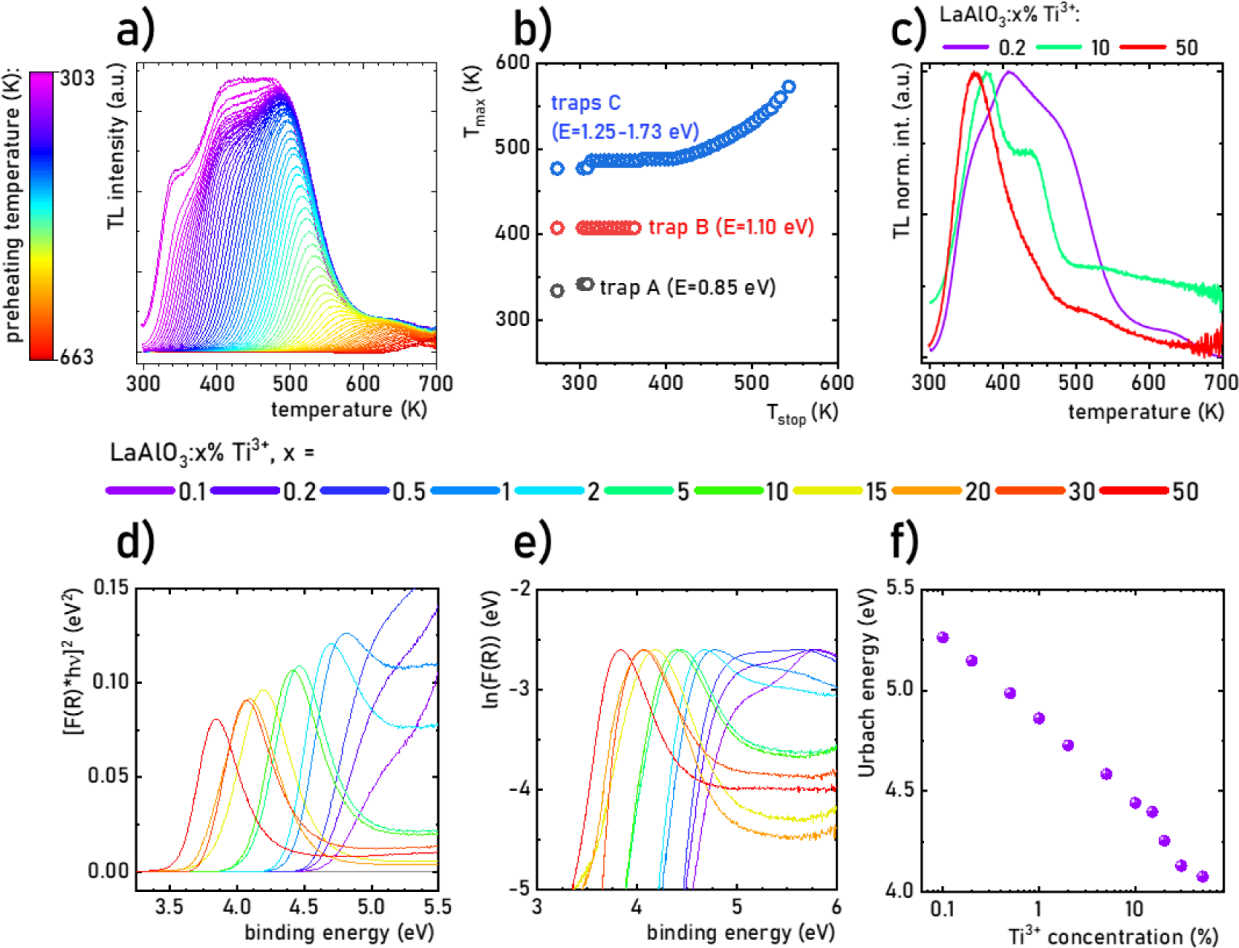
Influence of the preheating temperature *T*_stop_ on the TL glow curves (a) and local temperature maxima determined from the glow curves (b) of LaAlO_3_:0.2% Ti^3+^ phosphor irradiated with X-ray; the influence of Ti^3+^ concentration on the TL glow curves irradiated with X-ray (30 kV, 0.5m, irradiation time 5 min) (c); Tauc plot (d), Urbach plot (e), and Urbach energy (f) for LaAlO_3_ doped with different concentration of Ti^3+^ ions.

To better understand the mechanism of TL in the phosphors, we also investigated the heating-rate-dependent TL curves. In the 0.2% Ti^3+^ material, when the heating rate increases from 3 to 10 K s^–1^, the three TL peaks vary differently. The two low-temperature peaks (340, 405 K) lose intensity with the increasing heating rate. This is linked to an increase in the intensity of the third TL peak (490 K). However, the total TL intensity does not change. Presumably, the higher population of electrons in the conduction band for higher heating rates causes some of them to be retrapped in the deepest traps (490 K) and, consequently, generate TL at higher temperatures at the expense of the TL at 340 and 405 K. For the higher Ti^3+^ concentrations, the TL intensity decreases by about 13% (10% of Ti^3+^) and ∼20% (50% of Ti^3+^) with the increasing heating rate. In particular, the decrease is mostly due to the lessened TL related to the 340 K peak. Fading also differentiates the materials to some extent (Figure S19c, g, k). In the diluted system (0.2% Ti^3+^), all the TL peaks forming the broad TL band from ∼300 to about 580 K continuously lose their intensities over time. The most profound loss is observed for the lowest-temperature peak. It disappears within 30–60 min after charging. The next two peaks, at about 405 and 490 K, lose about 50% and 45% of their intensities after 300 min, respectively. Around 45 min after irradiation, the persistent luminescence decay becomes virtually exponential, especially for the 0.2 and 10% samples (Figure S19 d, h, l). This confirms the first-order character of the TL kinetics. To learn more about the properties of the traps, a series of TL glow curves were measured, taking advantage of the so-called partial cleaning or *T*_max_*–T*_stop_ experiments^83^ for the 0.2% sample, and the results are presented in Figure 5a. With increasing *T*_stop_, the glow curves become narrower as the intensity of their low-temperature part decreases. From the *T*_max_*–T*_stop_ plots (Figure 5b), it appears that the *T*_max_ of trap *C* (490 K, blue dots) increases almost linearly with *T*_stop_ which is characteristic of the continuous distribution of trap energies. Traps *A* and *B*, which give TL values at 340 and 405 K, appear to be individual trapping centers. Comparing the normalized glow curves of the three materials (Figure 5c), it is clear that their maxima shift toward lower temperatures with an increasing Ti^3+^ concentration. This comparison shows that with increasing Ti^3+^ concentration, the shallowest trap, giving TL around 340 K, contributes relatively more effectively to TL (and so to carrier trapping) compared to the deeper ones. Careful examination of the normalized TL glow curves (Figure 5c) allows us to note that especially the 340 and 405 K peaks move by a few degrees to lower temperatures with increasing Ti^3+^ concentration. This translates to the trap depths changing from 0.85 eV (0.2% Ti^3+^) to 0.80 eV (50% Ti^3+^) (trap *A*) and from 1.1 to 0.95 eV (trap *B*), respectively. The situation for trap *C*, due to the continuous distribution of energies, is more vague. It can be estimated that the energies of the traps span the 1.25–1.73 eV range.

Since the occurrence of persistent luminescence in the LaAlO_3_ structure is well-known in the literature, it is possible to compare theoretical calculations considering several categories of traps with experimental data (Figure S20). Among the lattice defects, Xiong et al.^35^ mainly mention 1) interstitial oxygen, 2) oxygen vacancy, and 3) creation of antisites, i.e., locating Al^3+^ ions in La^3+^ positions. The shallowest traps determined by the TL study energetically correspond to different kinds of oxygen vacancies *V*_*O*_^*2+*^, *V*_*O*_^*+*^, and *V*_*O*_^*0*^ and oxygen interstitial *I*_*O*_^*+*^, respectively. The most significant contribution of shallow traps at 360–400 K (traps A and B) for all samples is in agreement with the authors’ suggestion of the crucial importance of oxygen vacancies over other defects. Moreover, the energy of the deepest traps (traps *C*) corresponds to that of *I*_*O*_^*0*^. These two types of traps are considered the most probable, both based on theoretical considerations and determined in another work by electron spin resonance analysis.^85^ In the case of antisites, the traps could be located too deep to experimentally confirm the trap levels. However, Xiong et al.^35^ suggest that the occurrence of such states should not be expected. The Rietveld refinement of the XRPD patterns also does not confirm the presence of antisites in the investigated LaAlO_3_:Ti^3+^.

### Calculation of Optical Bandgap

To correctly interpret the interaction of electron traps with Ti^3+^ ions, it is necessary to determine the optical bandgap. For this reason, extinction spectra for LaAlO_3_:Ti^3+^ were measured (Figure S21, presented as a Kubelka–Munk transformation in Figure S22).^65^ It can be clearly seen that an absorption edge of the undoped LaAlO_3_ host material is separable for 0.1% Ti^3+^ from an additional band associated with intrabandgap states above ∼240 nm.^65^ The intensity of this band increases with respect to the absorption edge with a Ti^3+^ concentration up to 0.5% and then undergoes a gradual spectral red-shift to 322 nm for 50% Ti^3+^. Figure 5d shows the effect of Ti^3+^ concentration on the shape of the Tauc plot (wider spectral range in Figure S23). Plotting different variants of (*F*(*R*).*hv*)*^m^* for , , 2, and 3 determining the nature of the transition within the optical bandgap, Ye et al.^63^ and Liu et al.^86^ found that *m* = 2 was most likely, characterizing the transition as directly allowed. Due to the marginal effect of the overlapped band on the shape of the absorption edge for the LaAlO_3_:0.1% Ti^3+^ sample, an optical bandgap of 5.2 eV was determined. This is lower than the value reported in many articles for bulk crystalline materials or amorphous thin films (5.6–6.5 eV),^47,87−91^ but it stays in great agreement with the values obtained for nano- or microcrystalline powders (5.0–5.35 eV).^43,62,63,86^ These values are summarized in Table S5 and suggest a direct correlation of the optical bandgap value with the rhombohedral (∼6.1 eV) and cubic (∼5.2 eV) structures of LaAlO_3_.

However, for higher concentrations of Ti^3+^ ions, the additional component on the Tauc plot shifts toward lower energies, preventing the correct determination of the absorption edge. Therefore, the optical bandgap for the LaAlO_3_:Ti^3+^ phosphors was considered to remain constant, while the intrabandgap band is associated with the distortion caused by the doping of LaAlO_3_ known as the so-called Urbach intrabandgap state.^65^ A similar shift of Urbach states in LaAlO_3_ was observed with increasing concentrations of Mn^2+/3+^,^92^ Mg^2+^,^62^ and Ca^2+^/Mn^2+^,^64^ dopant ions, as well as Sr^2+^/Co^2+/3+^ and Sr^2+^/Mn^2+/3+^.^86^ The energy of the intrabandgap state, determined from the Urbach plot, is almost linear with an exponential increase in Ti^3+^ ion concentration from 5.2 to 4.1 eV (Figure 5e,f). It is expected that Urbach states may participate in the energy transfer between the electron traps and the conduction band.^93−95^ This is particularly probable since shallow traps associated with oxygen vacancies play a key role in the TL performance of LaAlO_3_:Ti^3+^ phosphors. Thus, a change in the energy of the Urbach states has a direct effect on the probability of such a transfer.

### VRBE Diagram

**Figure 6:**
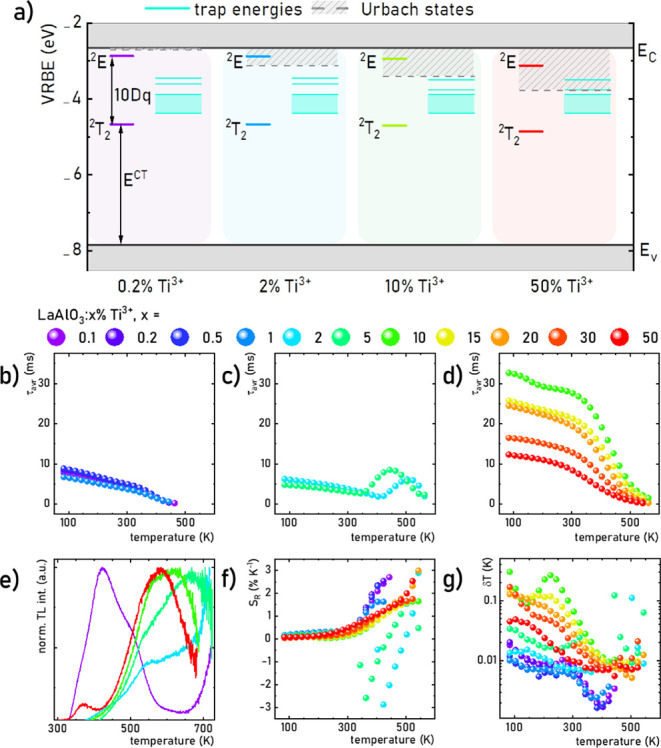
Influence of Ti^3+^ concentration on the VRBE diagram of LaAlO_3_:Ti^3+^ (a); thermal evolution of τ_avr_ of ^2^E(Ti^3+^) in LaAlO_3_ doped with 0.1–1% Ti^3+^ (b), 2–5% Ti^3+^ (c), and 10–50% Ti^3+^ (d); the influence of Ti^3+^ concentration on the TL glow curves irradiated with λ_exc_ = 365 nm (5 min) (e); thermal evolution of *S*_R_(τ_avr_) (g) of LaAlO_3_:Ti^3+^ phosphors.

The calculation of the energy gap, Urbach state energies, and electron trap energies under the conduction band allowed the construction of vacuum-referred binding energy (VRBE) diagrams for LaAlO_3_:Ti^3+^ in accordance with the instructions provided by Rogers and Dorenbos^81^ (Figure 6a). The Coulomb repulsion energy and the charge transfer transition for Eu^2+/3+^ in the LaAlO_3_ host material were used from refs^96,97^ (Figure S24). At first, the small energy distance between the ^2^E state and the conduction band, especially for 0.2% Ti^3+^, suggests a relatively high probability of energy transfer from the ^2^E state to the conduction band. This is clearly a smaller energy distance in comparison to other Ti^3+^-doped aluminate materials, such as Al_2_O_3_:Ti^3+^, YAlO_3_:Ti^3+^, and Y_3_Al_5_O_12_:Ti^3+^ (Figure S25).^54,55^ This may explain the elongated kinetics of Ti^3+^ ions luminescence observed already at low Ti^3+^ concentrations relative to counterparts in other host materials.^54−59,81^ Comparison of VRBE diagrams for different Ti^3+^ concentrations confirms that the position of ^2^E and ^2^T_2_ levels as well as electron traps varies slightly with Ti^3+^ concentration. However, the position of the Urbach states is crucial since they can play the role of an energy bridge between electron traps and the conduction band but also between the conduction band and the ^2^E state. This indicates the need to distinguish Urbach states as deformation of *d* states of La^3+^ ions located in the vicinity of Ti^3+^ ions and electron traps in the conduction band. This is clearly indicated for 2% and 10% Ti^3+^ ions. These two processes may positively influence the elongation of lifetimes of Ti^3+^ due to the enhanced probability of energy transfer from deeper electron traps to ^2^E via the Urbach states. This hypothesis could explain the origin of the ∼7-fold elongation of Ti^3+^ lifetimes above 10% of the dopant. Moreover, this mechanism may also lead to an intensity enhancement of luminescence from the ^2^E level, as confirmed by an order of magnitude increase in QY when the Ti^3+^ concentration increased from 5% to 10%.

Another important issue that can be derived from the VRBE diagram is the shape of the Ti^3+^ emission band. As indicated in Figure 2d, the emission band is strongly modified for Ti^3+^ concentrations lower than 5%. According to the position of Ti^3+^ energy levels and electron traps, it is possible to consider a parasitic energy transfer from the ^2^E level to the traps, interfering with the Gaussian distribution shape of ^2^E and ^2^T_2_ wave functions. This effect gradually weakens as the concentration of Ti^3+^ ions increases and disappears completely for 30% and 50% when the Urbach states are energetically below the part of electron traps, resulting in the quasi-Gaussian shape of the emission band expected for the spin-allowed ^2^E → ^2^T_2_ transition.

### Thermometric Performance of Ti^3+^ Luminescent Kinetics

The presence of electron traps and Urbach states in LaAlO_3_:Ti^3+^ also has a significant effect on the thermometric performance of τ_avr_ of ^2^E(Ti^3+^) (Figures 6b–e). According to the previously presented τ_avr_ at 83 K, these values can be divided into two groups: for 0.1–5% and 10–50% of Ti^3+^ ions. For the first group, a gradual thermal shortening of the kinetics can be determined up to 363 K, beyond which the thermal quenching rate further increases for samples with Ti^3+^ content spanning 0.1–1% (Figure 6b, see also Figures S26 and S27), while τ_avr_ increases above 403 and 363 K for LaAlO_3_:2% Ti^3+^ and LaAlO_3_:5% Ti^3+^, respectively. This may suggest gradual thermal feeding of the ^2^E level from electron traps through the conduction band or through a local migration of carriers over an energy barrier for the 2 and 5% samples (Figure 6a). This is consistent with the gradual shifting of the TL band, which starts growing above 650 K for LaAlO_3_:0.2% Ti^3+^ and LaAlO_3_:2% Ti^3+^ when charged with 365 nm radiation and is well observed for LaAlO_3_ doped with 5, 10, and 50% of Ti^3+^ (Figure 6e). Unfortunately, it was impossible to record this band above 700 K due to the increasing contribution of blackbody radiation in the TL measurements. Beyond 10% of Ti^3+^ concentration, the decrease of temperature at which the probability of thermal quenching of τ_avr_ of ^2^E(Ti^3+^) increases is noted from 323 to 263 K for 10% and 50% Ti^3+^, respectively (Figure 6d). Therefore, despite the presence of two energy transfer mechanisms between the traps and the conduction band, facilitated by Urbach states followed by a transition to the ^2^E state, further reduction of the energy of the ^2^E and ^2^T_2_ levels with increasing Ti^3+^ content and concentration quenching facilitates depopulation of the ^2^E state. This is also the reason that the lifetimes are gradually reduced.

The thermally dependent Ti^3+^ luminescence kinetics reaching up to tens of microseconds is encouraging for applications in luminescence thermometry. To compare the thermal rate of τ_avr_ change with different Ti^3+^ concentrations, the relative sensitivity was determined (according to eq S1 and Figure 6f). For LaAlO_3_ doped with 0.1–50% of Ti^3+^, *S*_R_ values do not exceed 0.5% K^–1^ in the range 83–303 K. However, for samples with 0.1–1% of Ti^3+^, *S*_R_ increased gradually above 303 K, reaching maximum values for the lowest τ_avr_ value, i.e., *S*_Rmax_ = 2.70% K^–1^ at 443 K for 0.1% Ti^3+^. A similar monotonic increase in *S*_R_ was observed for 10–50% of the Ti^3+^ ions. However, the increase in *S*_R_ was less spectacular and was over a wider temperature range. A maximum value of *S*_R_ = 2.99% K^–1^ at 543 K was calculated for 15% Ti^3+^. Due to the observed increase in τ_avr_ values for 2% and 5% Ti^3+^, *S*_R_ values also reached significant negative values, i.e., *S*_R_ = −2.87% K^–1^ at 423 K and *S*_R_ = −2.58% K^–1^ at 363 K for 2% and 5%, respectively. Relatively high τ_avr_ and *S*_R_ values exceeding 1.0% K^–1^ provided low-temperature uncertainty δ*T* (eq S2 and Figure 6g). Their value did not exceed 0.3 K throughout the temperature range, while over 300 K δ*T* < 0.03 K was obtained for all phosphors analyzed. This allows us to conclude that the best thermometric performance of the lifetime-based LaAlO_3_:Ti^3+^ luminescence thermometer can be found in the 300–500 K range, which appears to be the most relevant for applications in many fields.

## Conclusions

In this work, the luminescent properties of Ti^3+^ ions in a wide range of concentrations (up to 50%) in LaAlO_3_ were investigated to understand and describe the mechanisms that lead to the occurrence and elongation of persistent luminescence dependent on the content of Ti^3+^ ions as the dopant. In the case of LaAlO_3_:Ti^3+^ with low Ti^3+^ concentration (below 5%), the luminescence kinetics of the ^2^E state were found to be several times longer than expected for Ti^3+^ ions. This effect was enhanced when the concentration of Ti^3+^ exceeded 10%, reaching τ_avr_ = 32.36 ms at 83 K and 30.98 ms at 298 K. The origin of this behavior was shown to be related to the presence of several types of electron traps in LaAlO_3_:Ti^3+^, resulting from oxygen vacancies and interstitial oxygen, and the location of the ^2^E level close to the conduction band, allowing energy transfer from electron traps to Ti^3+^ ions. As the Ti^3+^ concentration increased, the effect of Urbach states on this transfer increased, enabling the activation of deeper traps. The analysis of results presented in this work allows for control of the kinetics of Ti^3+^ persistent luminescence through dopant-induced defect engineering. Finally, the potential of LaAlO_3_:Ti^3+^ for lifetime-based thermometry was analyzed, indicating that, depending on the chosen Ti^3+^ ion concentration, it is possible to achieve *S*_R_ > 1% K^–1^ in the range of 300–500 K.

## Supporting Information Available

Results of additional XRPD and ICP-EOS measurements; Rietveld refinement; representative TEM images; excitation spectra and luminescence decay profiles as a function of the annealing temperature and a function of Ti^3+^ concentration; comparison of Ti^3+^-doped phosphors reported in the literature; comparison of Ti^3+^ and Cr^3+^ emission spectra and luminescence decay profiles in LaAlO_3_; TRES map as a function of λ_exc_; comprehensive thermoluminescence study on LaAlO_3_:Ti^3+^; Kubelka–Munk transformation absorption spectra and Tauc plot; comparison of optical bandgap values reported for LaAlO_3_ materials from the literature; thermal evolution of emission spectra of LaAlO_3_:Ti^3+^; equations for the relative sensitivity and temperature determination uncertainty (PDF)

## Abbreviations

CT: charge transfer
ICP-OES: inductively coupled plasma optical emission spectroscopy
QY: quantum yield
TEM: transmission electron microscope
TL: thermoluminescence
TRES: time-resolved emission/excitation spectroscopy
XRPD: X-ray powder diffraction
VRBE: vacuum-referred binding energy
