# Supplementary material for: Understanding the Influence of Electron Traps and Urbach States on the Kinetics of Ti3+ Persistent Luminescence in LaAlO3:Ti3+
Source: Inorg Chem. 2025 Feb 7;64(6):3137–49. doi: 10.1021/acs.inorgchem.5c00390 (PMC11836934; doi:10.1021/acs.inorgchem.5c00390)
Supplement: Supplementary file 1 — ic5c00390_si_001.pdf [file ic5c00390_si_001.pdf]

# Supporting Information

# Understanding the influence of electron traps and Urbach states on the kinetics of $Ti^{3+}$ persistent luminescence in $LaAlO_3:Ti^{3+}$

*Wojciech M. Piotrowski[1*], Justyna Zeler[2], Vasyl Kinzhybalo[1], Karolina Ledwa[1], Paulina Bukowska[2], Eugeniusz Zych[2], Lukasz Marciniak[1]*

[1]Institute of Low Temperature and Structure Research, Polish Academy of Sciences, Okólna 2, 50-422 Wroclaw, Poland

[2]Faculty of Chemistry, University of Wroclaw, 14.p F. Joliot-Curie Street, PL-50383, Wroclaw, Poland

KEYWORDS luminescence thermometry, titanium ions; excited state lifetime; lanthanum-aluminum perovskites

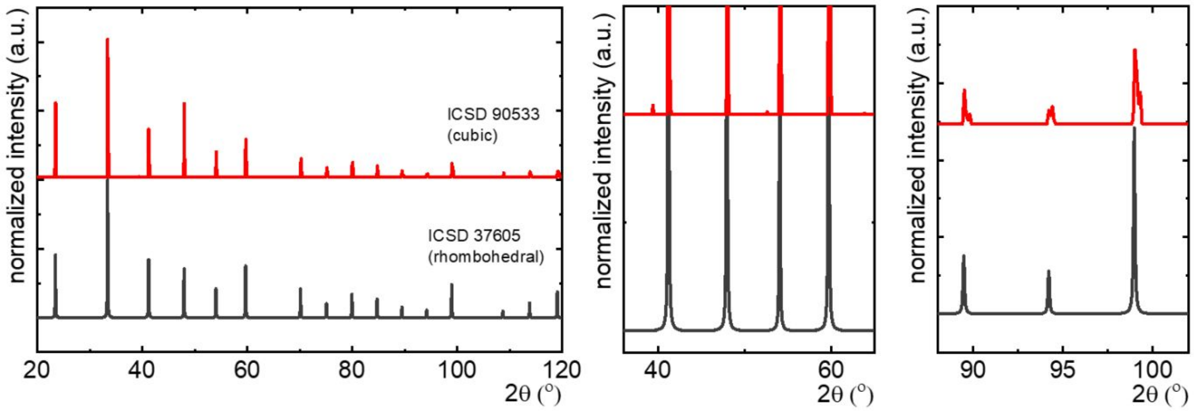


Figure S1.The comparison of XRPD reference patterns for cubic and rhombohedral structures of $LaAlO_3$.

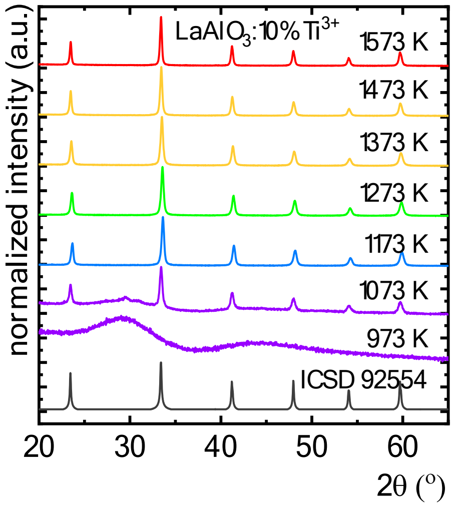


Figure S2.The XRPD patterns of $LaAlO_3$:10% $Ti^{3+}$ powders annealed at different temperatures.

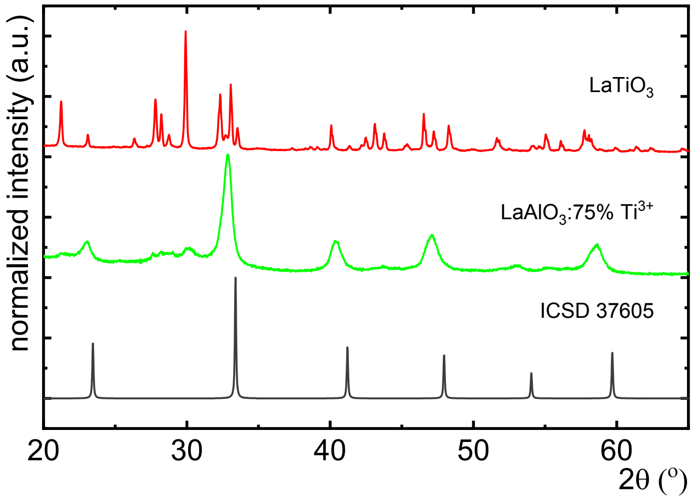


Figure S3.The XRPD patterns of $LaAlO_3$:75% $Ti^{3+}$ and $LaTiO_3$.

Table S1. Unit cell parameters calculated with Rietveld refinement for $LaAlO_3$:x% $Ti^{3+}$ phosphors.

| sample | a (Å) | V (Å$^3$) | R profile (%) | weighted R profile (%) | goodness of fit |
|---|---|---|---|---|---|
| $LaAlO_3$:0.1% $Ti^{3+}$ | 3.791 | 54.497 | 2.19 | 2.82 | 7.84 |
| $LaAlO_3$:0.2% $Ti^{3+}$ | 3.791 | 54.514 | 1.92 | 2.65 | 6.91 |
| $LaAlO_3$:0.5% $Ti^{3+}$ | 3.791 | 54.506 | 2.04 | 2.76 | 7.48 |
| $LaAlO_3$:1% $Ti^{3+}$ | 3.793 | 54.571 | 2.10 | 2.91 | 7.78 |
| $LaAlO_3$:2% $Ti^{3+}$ | 3.795 | 54.659 | 2.30 | 3.21 | 10.04 |
| $LaAlO_3$:5% $Ti^{3+}$ | 3.798 | 54.798 | 2.19 | 2.98 | 8.73 |
| $LaAlO_3$:10% $Ti^{3+}$ | 3.804 | 55.058 | 2.35 | 3.17 | 8.80 |
| $LaAlO_3$:15% $Ti^{3+}$ | 3.808 | 55.238 | 2.20 | 2.98 | 8.33 |
| $LaAlO_3$:20% $Ti^{3+}$ | 3.812 | 55.416 | 2.62 | 3.56 | 10.99 |
| $LaAlO_3$:30% $Ti^{3+}$ | 3.821 | 55.787 | 2.15 | 3.14 | 9.05 |
| $LaAlO_3$:50% $Ti^{3+}$ | 3.834 | 56.388 | 3.10 | 4.26 | 15.92 |

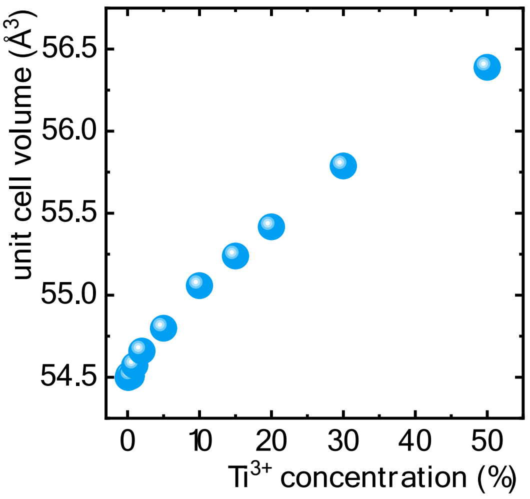


Figure S4. Influence of $Ti^{3+}$ concentration on unit cell volume in $LaAlO_3$:x% $Ti^{3+}$ phosphors.

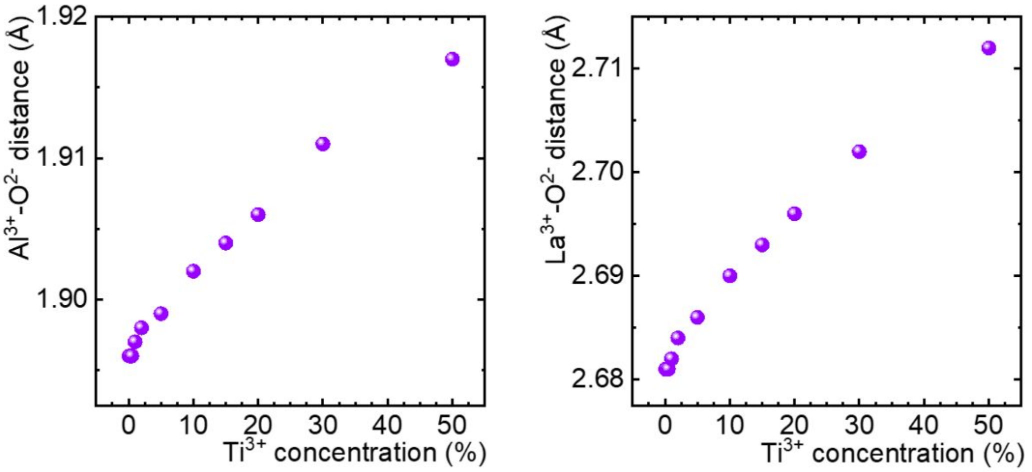


Figure S5. Influence of $Ti^{3+}$ concentration on $Al^{3+}$-$O^{2-}$ and $La^{3+}$-$O^{2-}$ distances in $LaAlO_3$:x% $Ti^{3+}$ phosphors.

Table S2. Results of ICP-EOS measurements for $LaAlO_3$:x% $Ti^{3+}$ phosphors.

| sample | Concentration in % (in respect to the $La^{3+}$ as 100%) | |
|---|---|---|
| | $Al^{3+}$ | $Ti^{3+}$ |
| $LaAlO_3$:0.1% $Ti^{3+}$ | 98.67 | 0.21 |
| $LaAlO_3$:0.2% $Ti^{3+}$ | 100.07 | 0.23 |
| $LaAlO_3$:0.5% $Ti^{3+}$ | 98.44 | 0.40 |
| $LaAlO_3$:1% $Ti^{3+}$ | 99.66 | 1.10 |
| $LaAlO_3$:2% $Ti^{3+}$ | 97.86 | 2.40 |
| $LaAlO_3$:5% $Ti^{3+}$ | 91.40 | 5.71 |
| $LaAlO_3$:10% $Ti^{3+}$ | 90.21 | 11.43 |
| $LaAlO_3$:15% $Ti^{3+}$ | 87.11 | 17.27 |
| $LaAlO_3$:20% $Ti^{3+}$ | 80.95 | 22.71 |
| $LaAlO_3$:30% $Ti^{3+}$ | 71.46 | 34.16 |
| $LaAlO_3$:50% $Ti^{3+}$ | 50.56 | 55.04 |

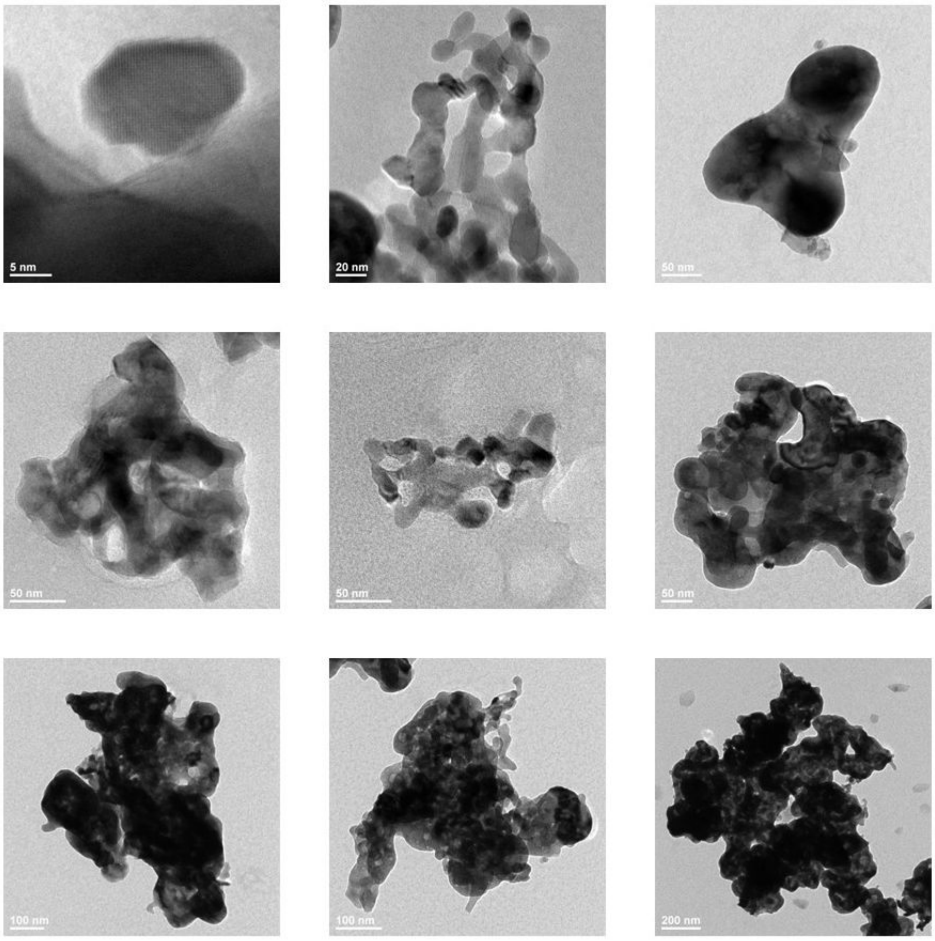


Figure S6. The representative TEM image for $LaAlO_3$:0.2% $Ti^{3+}$ sample.

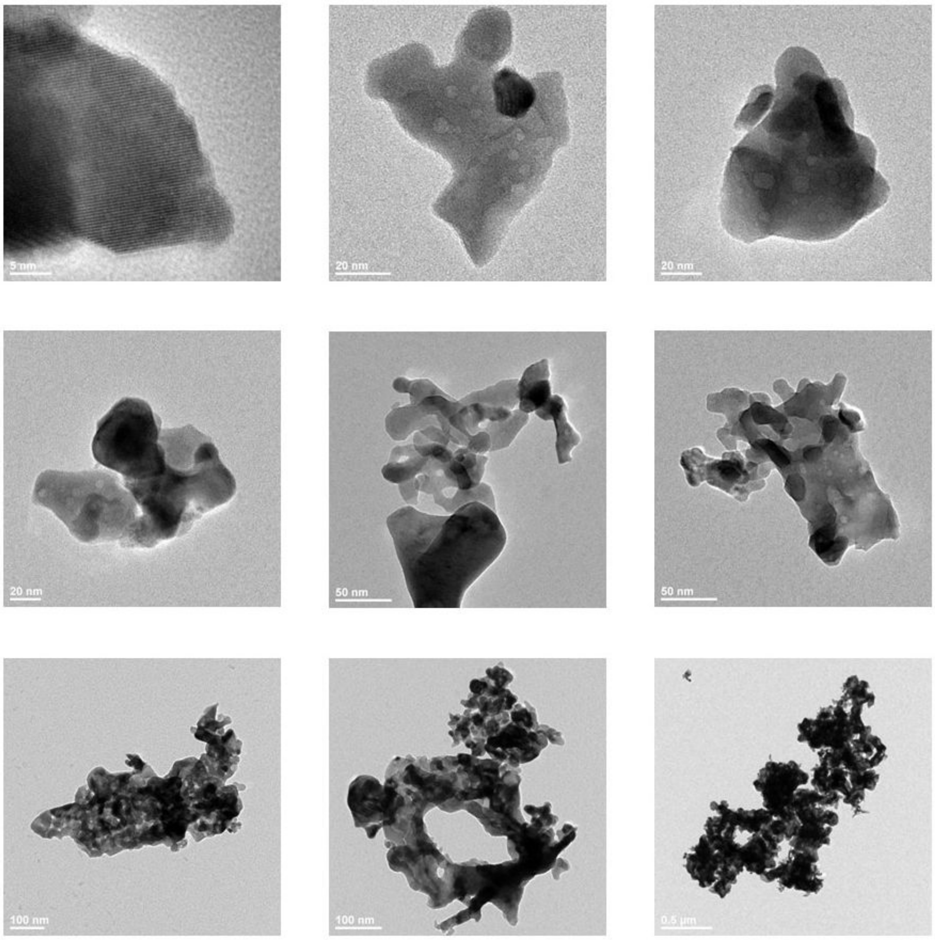


Figure S7. The representative TEM image for $LaAlO_3$:10% $Ti^{3+}$ sample.

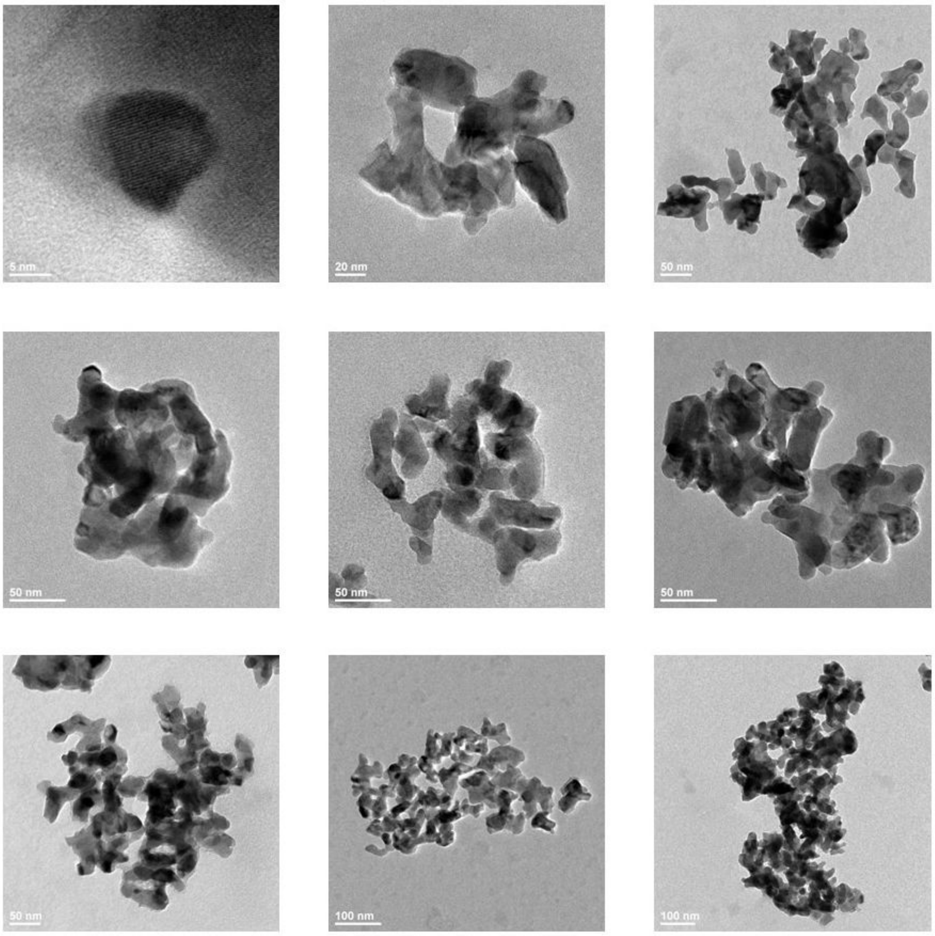

Figure S8. The representative TEM image for $LaAlO_3$:50% $Ti^{3+}$ sample.

## *Luminescent properties characterization*

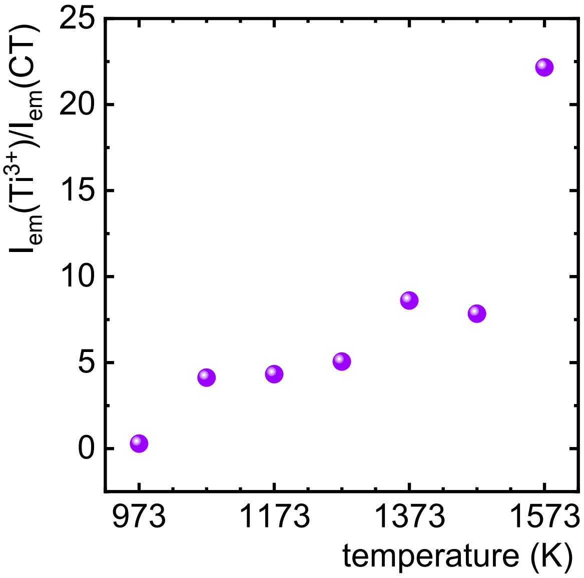

Figure S9. Influence of annealing temperature on the intensity ratio of $^2E \rightarrow {}^2T_2$ and CT emission bands in $LaAlO_3$:10% $Ti^{3+}$ phosphors.

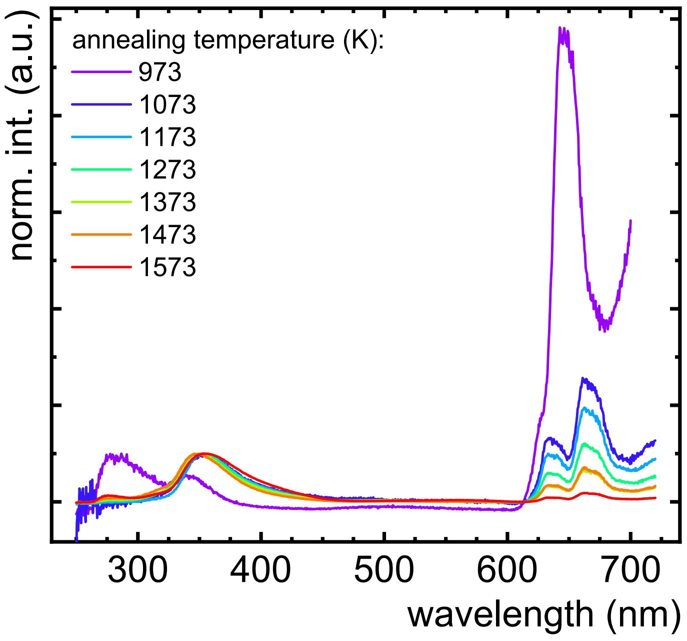


Figure S10. Influence of annealing temperature on excitation spectra measured at 83K in $LaAlO_3$:10% $Ti^{3+}$ phosphors.

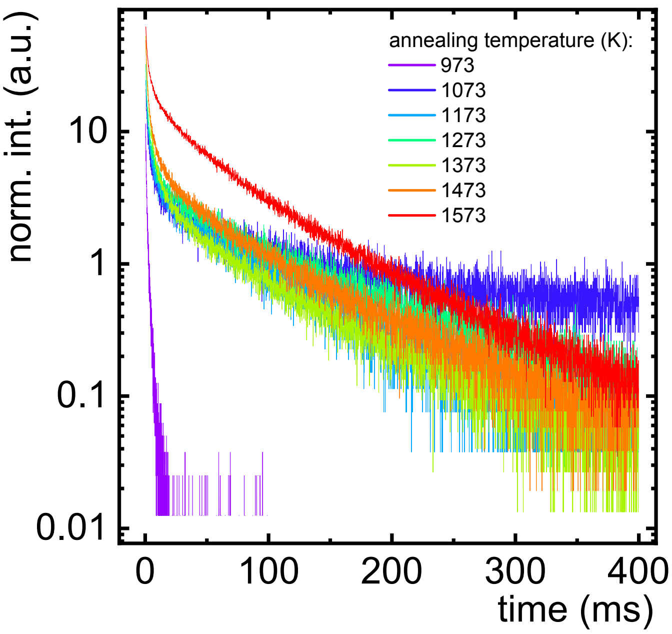


Figure S11. Influence of annealing temperature on the luminescence decay profiles for $^2E(Ti^{3+})$ state measured at 83K in $LaAlO_3$:10% $Ti^{3+}$ phosphors ($\lambda_{exc}$=351nm, $\lambda_{em}$=742nm).

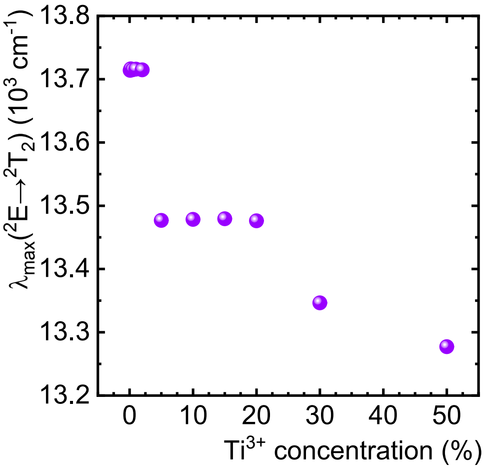


Figure S12. Influence of $Ti^{3+}$ concentration on the position of $^2E \rightarrow ^2T_2$ emission band in $LaAlO_3:Ti^{3+}$ phosphors.

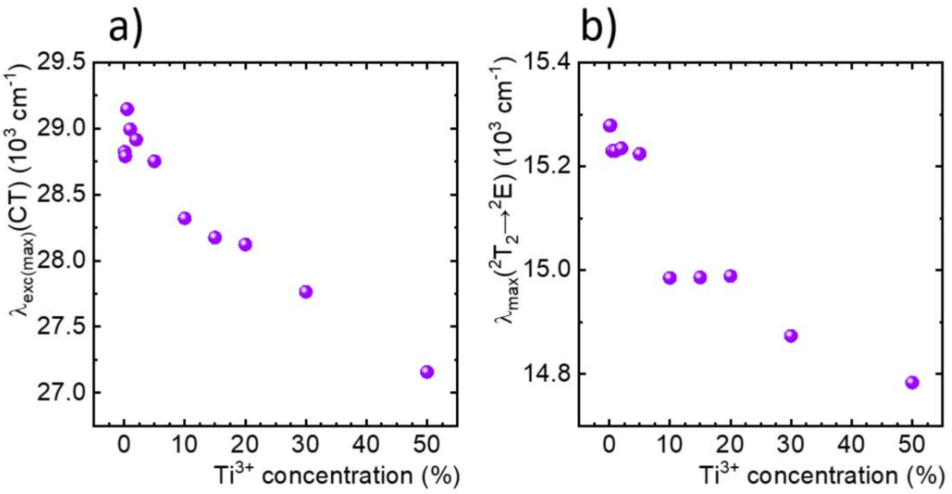


Figure S13. Influence of $Ti^{3+}$ concentration on the position of CT excitation band – a) and $^2T_2 \rightarrow ^2E$ excitation band – b) in $LaAlO_3:Ti^{3+}$ phosphors.

Table S3. Comparison of energy of excitation and emission spectra for $Ti^{3+}$ doped phosphors.

| | $LaAlO_3$ | Y3Al5O12 [1] | $BeAl_2O_4$ [2] | $Al_2O_3$ [3] | $Al_2O_3$ [1] | YAlO3 [1] |
|---|---|---|---|---|---|---|
| $E_{exc}$ ($cm^{-1}$) | 15278 | 16700 | 17440 | 17720 | 18200 | 20400 |
| $E_{em}$ ($cm^{-1}$) | 13714 | 13300 | 15870 | 14200 | 13500 | 16400 |
| 10Dq ($cm^{-1}$) | 14495 | 18350 | - | 1913 | 19300 | 21950 |
| ΔE ($cm^{-1}$) | 1564 | 3400 | 1570 | 3520 | 4700 | 4000 |
| ΔE (eV) | 0.20 | 0.42 | 0.20 | 0.43 | 0.58 | 0.50 |

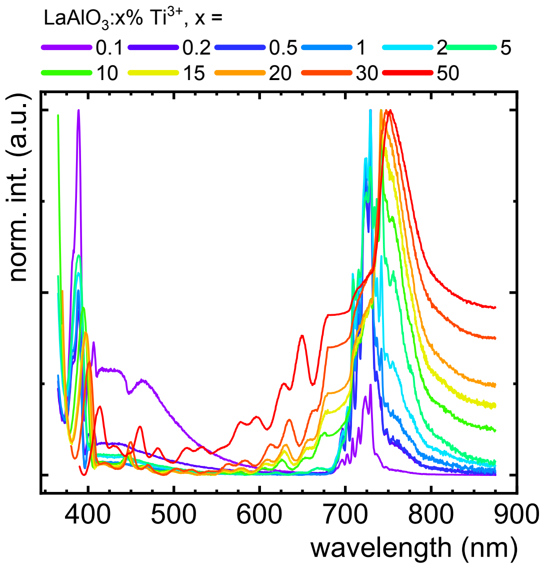


Figure S14. Influence of $Ti^{3+}$ concentration on the emission spectra in $LaAlO_3:Ti^{3+}$ phosphors (including CT emission band).

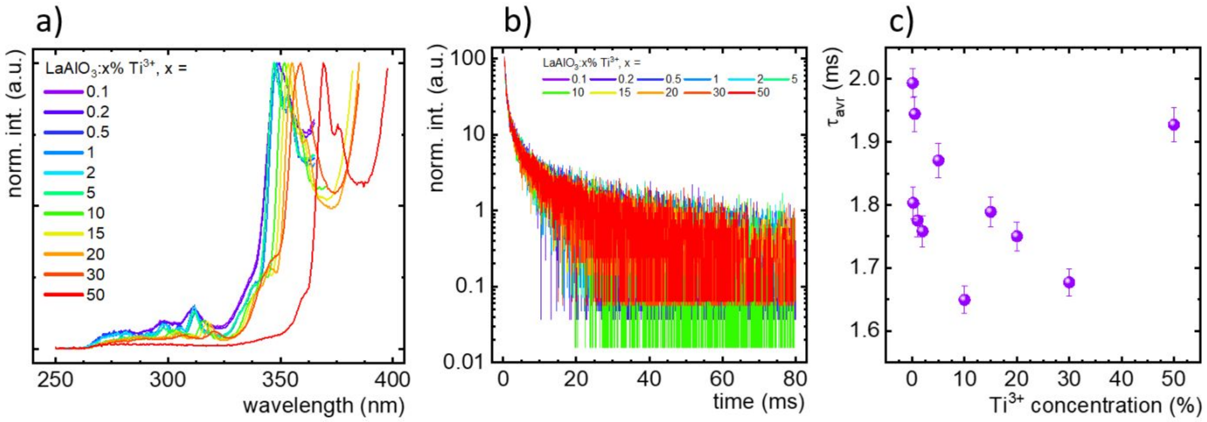


Figure S15. Influence of $Ti^{3+}$ concentration on the excitation spectra measured for CT emission band – a), luminescence decay profiles – b) and $\tau_{avr}$ of CT band – c) in $LaAlO_3:Ti^{3+}$ phosphors.

Table S4. The selected spectroscopic parameters of the representative $Ti^{3+}$-doped phosphors.

| host material | the maximum of excitation band (nm) | the maximum of emission band (nm) | $\tau_{avr}$ at 10 K (ms) | $\tau_{avr}$ at 293 K (ms) | ref. |
|---|---|---|---|---|---|
| $Al_2O_3$ | 490; 550 | 740 | ~$3.8\cdot10^{-3}$ | | 1 |
| $Al_2O_3$ | 487; 563 | 703; 788 | | | 3 |
| $YAlO_3$ | 425; 490 | 610 | $17\cdot10^{-3}$ | | 1 |
| $Y_3Al_5O_{12}$ | 500; 599 | 750 | $53\cdot10^{-3}$ | $2\cdot10^{-3}$ | 1 |
| $Y_3Al_5O_{12}$ ($Ti^{3+}/Ti^{4+}$) | | 820 | | 6 | 4 |
| $BeAl_2O_4$ | 500; 573 | 759 | ~$5.0\cdot10^{-3}$ | | 2 |
| $BeAl_6O_{10}$ | 475; 580 | 800 | | | 5 |
| $LaMgAl_{11}O_{19}$ | 570 | 775 | $6\cdot10^{-3}$ | $4\cdot10^{-3}$ | 6 |
| $SrTiO_3:La^{3+}$ | 370 ($Ti^{4+}$); 405 | 791 | 64.75 | - | 7 |
| $SrTiO_3:Gd^{3+}$ | 375($Ti^{4+}$); 410 | 791 | 20.55 | 0.191 | 8 |
| $CaTiO_3:Gd^{3+}$ | 335($Ti^{4+}$); 405 | 770 | 9.35 | 0.655 | 8 |
| $LaAlO_3$ (0.1% $Ti^{3+}$) | 343; 653 | 729 | 7.78 (83K) | 4.77 | this work |
| $LaAlO_3$ (10% $Ti^{3+}$) | 351; 663 | 741 | 32.36 (83K) | 27.57 | this work |

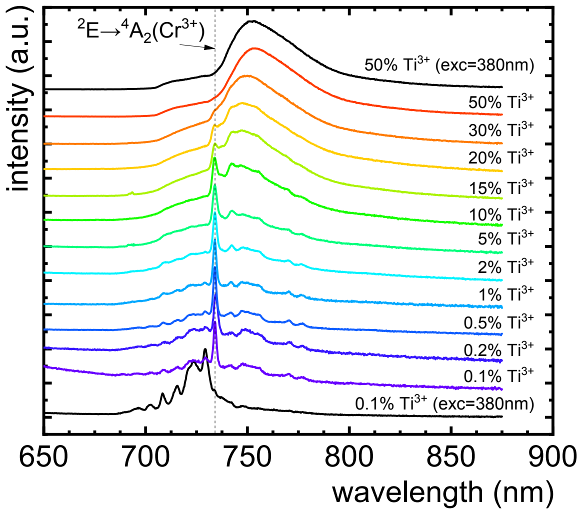


Figure S16. Comparison of the emission spectra measured with $\lambda_{exc}$=445nm (suitable for $Cr^{3+}$ ions) for different $Ti^{3+}$ concentration with the emission spectra measured for 0.1% $Ti^{3+}$ and 50% $Ti^{3+}$ with $\lambda_{exc}$=380nm (suitable for $Ti^{3+}$ ions) in $LaAlO_3:Ti^{3+}$ phosphors.

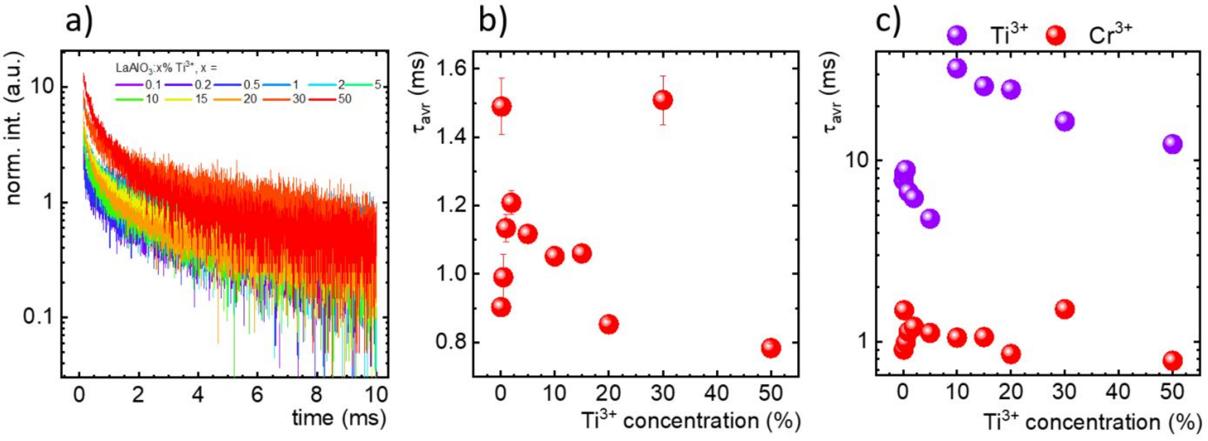


Figure S17. Influence of $Ti^{3+}$ concentration on the luminescence decay profiles – a) and $\tau_{avr}$ of $^2E(Cr^{3+})$ – b) in $LaAlO_3:Ti^{3+}$ phosphors; comparison of $\tau_{avr}$ of $^2E(Cr^{3+})$ and $^2E(Ti^{3+})$ as the function of $Ti^{3+}$ concentration – c).

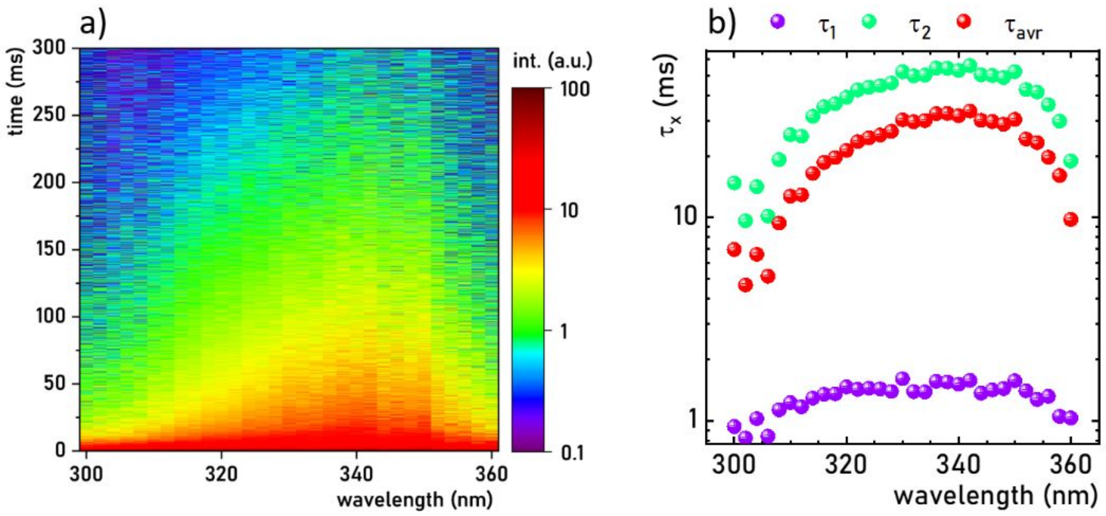


Figure S18. TRES map as a function of $\lambda_{exc}$ measured for $\lambda_{em}$=742nm for $LaAlO_3$:10% $Ti^{3+}$ phosphor.

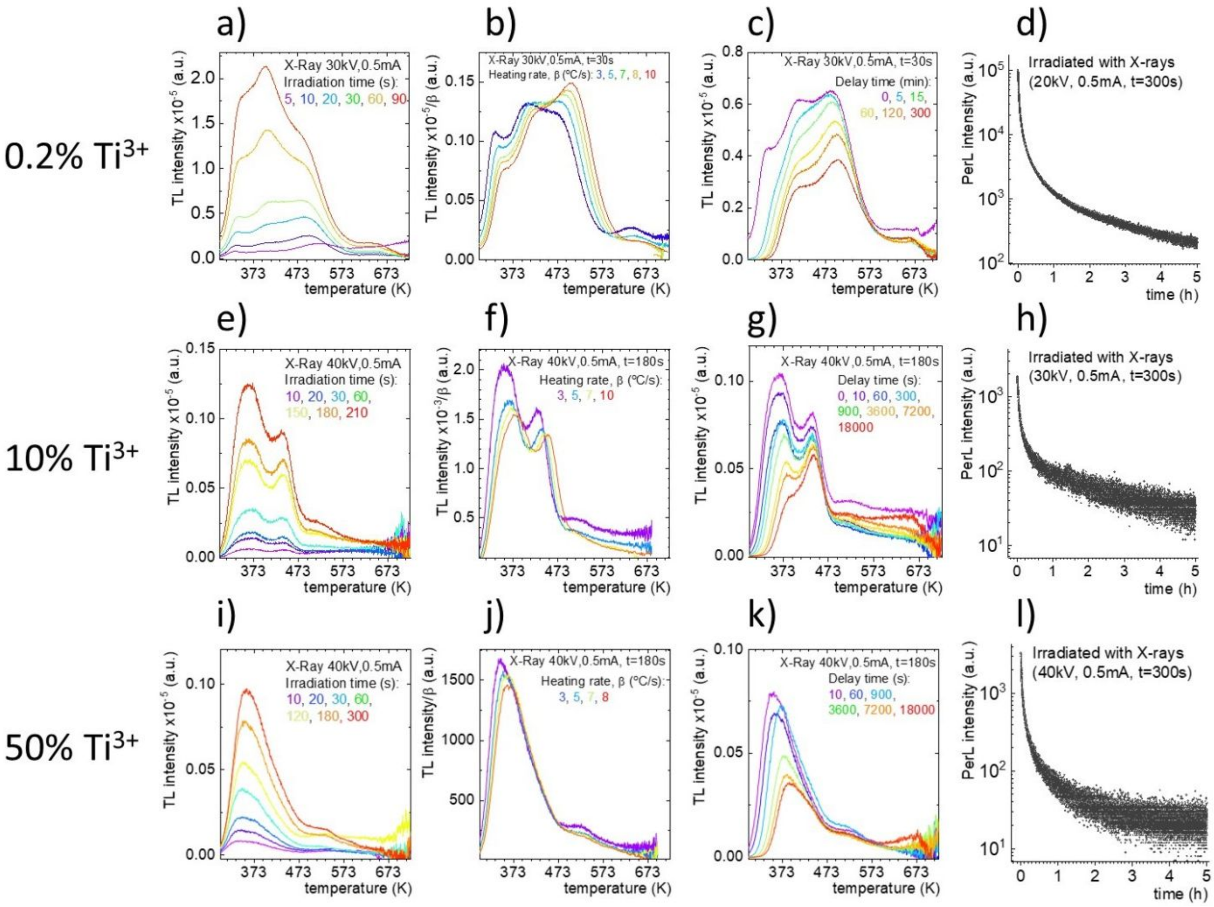


Figure S19. Comprehensive thermoluminescence study on $LaAlO_3:Ti^{3+}$ phosphors doped with 0.2% $Ti^{3+}$ ( a-d), 10% $Ti^{3+}$ (e-h) and 50% $Ti^{3+}$ (i-l): TL glow curves as a function of irradiation time (dose) with X-rays (a, e, i); TL glow curves as a function of the heating rate (b, f, j); TL glow curves as a function of delay time (c, g, k); PersL decay profiles (d, h, l).

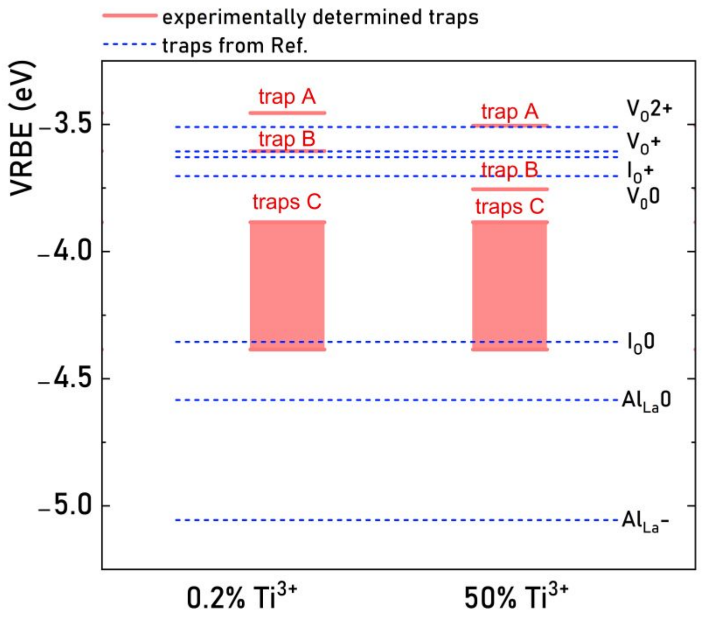


Figure S20. Comparison of trap depth of experimentally determined traps in $LaAlO_3:Ti^{3+}$ with theoretically calculated traps from Ref.[9]

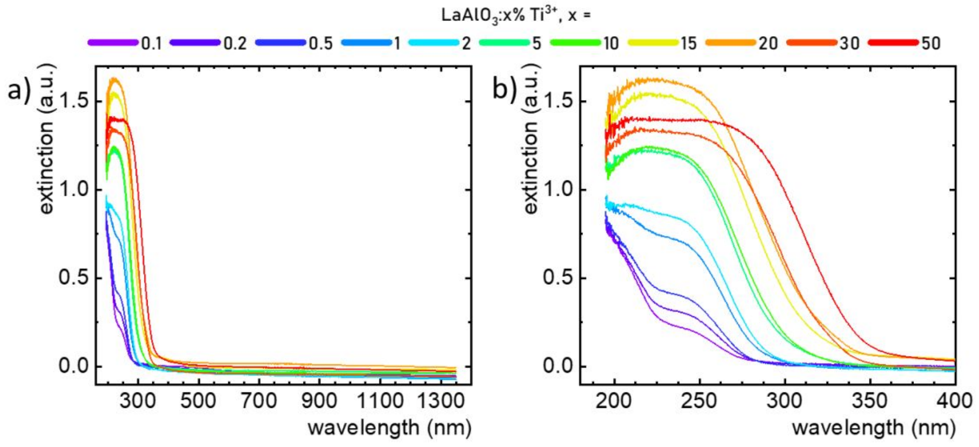


Figure S21. Influence of $Ti^{3+}$ concentration on the extinction spectra of $LaAlO_3$:$Ti^{3+}$ phosphors in the spectral range of 160-1400 nm – a), 180-400 nm – b).

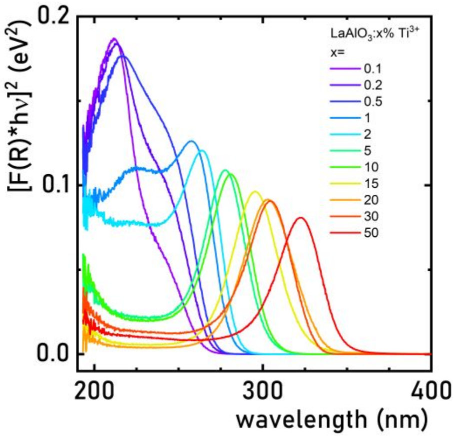


Figure S22. Kubelka-Munk transformation absorption spectra of $LaAlO_3$ doped with different concentration of $Ti^{3+}$ ions.

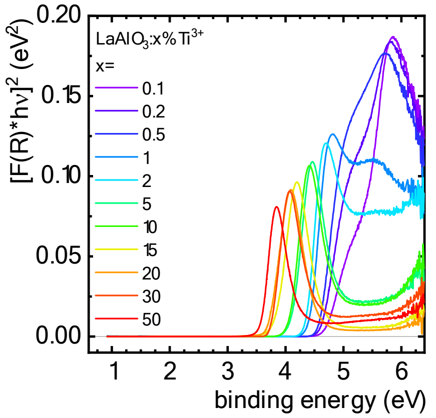


Figure S23. Tauc plot of $LaAlO_3$ doped with different concentration of $Ti^{3+}$ ions.

Table S5. Comparison of optical bandgap values reported for different $LaAlO_3$ materials.

| Optical bandgap (eV) | Comment | Ref. |
|---|---|---|
| 5.0 | polycrystalline sample, sol-gel method with annealing temperature 1573K | 10 |
| 5.18 | sol-gel method with annealing temperature 1573K | 11 |
| 5.20 | 30±10 nm, annealed at 1273K | this work |
| 5.35 | ~45–49 nm, combustion method with annealing temperature 1173K | 12 |
| 5.35 | 5–20 µm | 13 |
| 5.6 | d-state band gaps in bulk crystalline material | 14 |
| 5.6 | values obtained for crystalline material | 15 |
| 5.8 | amorphous film | 16 |
| 5.84 | values obtained for thicker films | 17 |
| 6.0 | d-state band gaps in bulk crystalline material | 14 |
| 6.2 | values obtained for amorphous film | 15 |
| 6.2 | amorphous film | 18 |
| 6.33 | values obtained for thinner films | 17 |
| 6.5 | thin film on silicon | 19 |

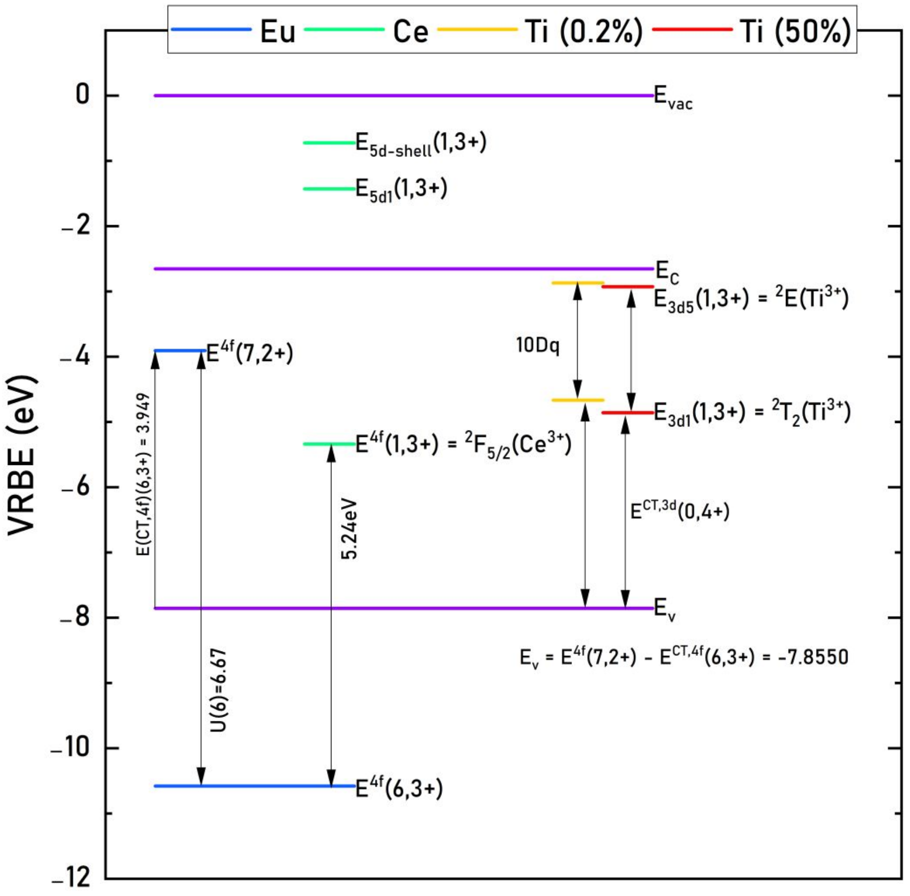


Figure S24. VRBE diagram for Eu, Ce and Ti ions in $LaAlO_3$.

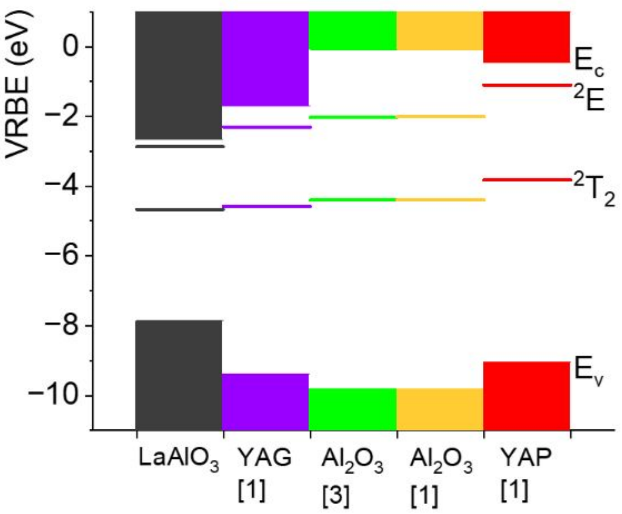


Figure S25. VRBE diagram for representative $Ti^{3+}$ doped aluminates [1,3].

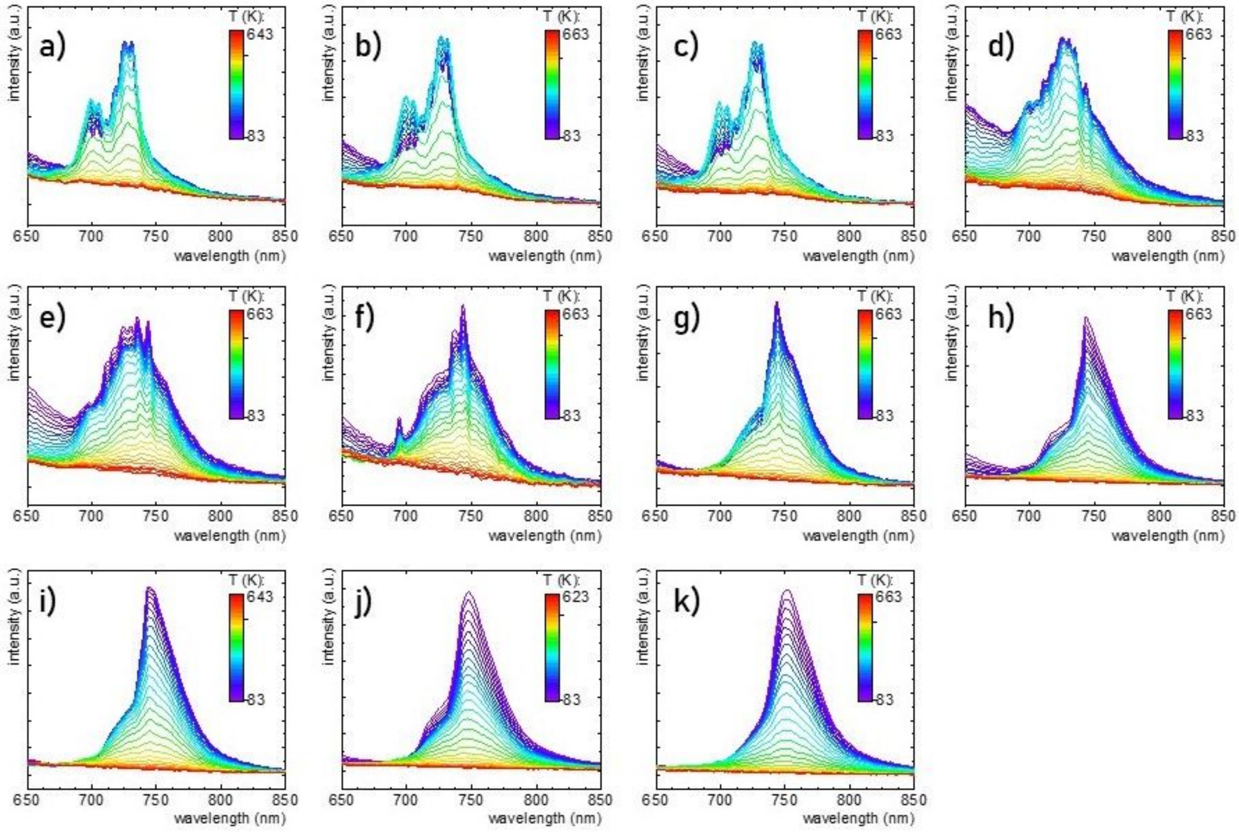


Figure S26. Thermal evolution of emission spectra of $LaAlO_3$:x% $Ti^{3+}$ phosphors, where x = 0.1 – a), 0.2 – b), 0.5 – c), 1 – d), 2 – e), 5 – f), 10 – g), 15 – h), 20 – i), 30 – j), 50 – k).

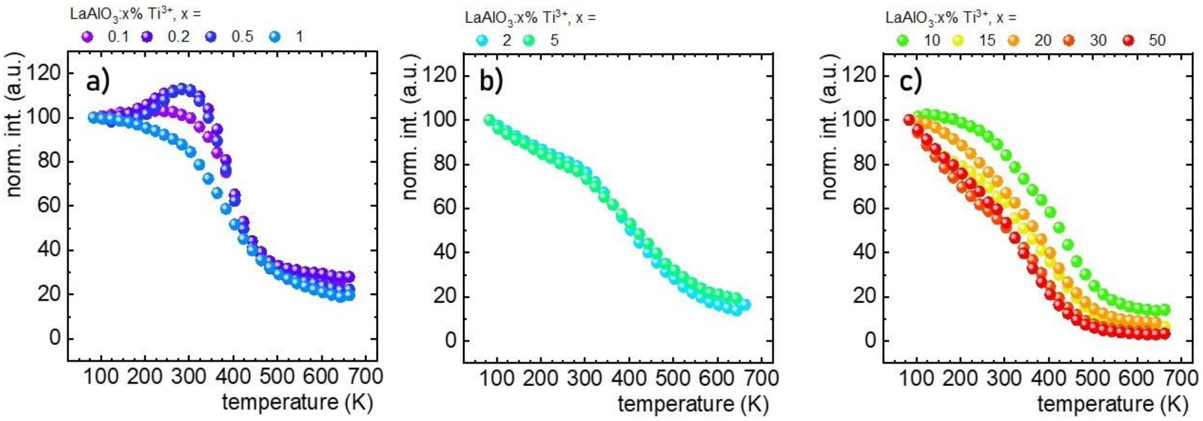

Figure S27. Thermal evolution of intensity of $^{2}E \rightarrow ^{2}T_{2}$ emission band in $LaAlO_3$:x% $Ti^{3+}$ phosphors.

The relative sensitivities of the potential thermometers based on $\tau_{avr}$ of $^{2}E(Ti^{3+})$ were determined, according to Eq. S1:

$$S_R = \frac{1}{\tau_{avr}} \frac{\Delta \tau_{avr}}{\Delta T} \times 100\% \qquad \text{(Eq. S1)}$$

where and $\Delta\tau_{avr}$ represents the change of $\tau_{avr}$for the $\Delta T$ change of temperature.

Temperature determination uncertainty for lifetime based approach was calculated using Eq. S2:

$$\delta T = \frac{1}{S_R} \cdot \frac{\delta \tau_{avr}}{\tau_{avr}} \qquad \text{(Eq. S2)}$$

Reference


(1) Yamaga, M.; Gao, Y.; Rasheed, F.; O'Donnell, K. P.; Henderson, B.; Cockayne, B. Radiative and Non-Radiative Decays from the Excited State of Ti3+ Ions in Oxide Crystals. *Applied Physics B 1990 51:5* **1990**, *51* (5), 329–335. https://doi.org/10.1007/BF00348969.

(2) Sugimoto, A.; Yamagishi, K.; Kim, P. H.; Namba, S.; Yamaguchi, Y.; Segawa, Y.; Anzai, Y. Spectroscopic Properties of $Ti^{3+}$-Doped $BeAl_2O_4$. *JOSA B, Vol. 6, Issue 12, pp. 2334-2337* **1989**, *6* (12), 2334–2337. https://doi.org/10.1364/JOSAB.6.002334.

(3) García-Revilla, S.; Rodríguez, F.; Valiente, R.; Pollnau, M. Optical Spectroscopy of Al 2 O 3 :Ti 3+ Single Crystal under Hydrostatic Pressure. The Influence on the Jahn-Teller Coupling. *J. Phys.: Condens. Matter* **2002**, *14*, 447–459.

(4) Drabik, J.; Cichy, B.; Marciniak, L. New Type of Nanocrystalline Luminescent Thermometers Based on Ti3+/Ti4+ and Ti4+/Ln3+ (Ln3+ = Nd3+, Eu3+, Dy3+) Luminescence Intensity Ratio. *Journal of Physical Chemistry C* **2018**. https://doi.org/10.1021/acs.jpcc.8b02328.

(5) Solntsev, V. P.; Pestryakov, E. v.; Alimpiev, A. I.; Tsvetkov, E. G.; Matrosov, V. N.; Trunov, V. I.; Petrov, V. v. BeAl6O10:Cr3+ (Ti3+, Ni2+) Laser Crystals and Their Spectroscopic Characteristics. *Opt Mater (Amst)* **2003**, *24* (3), 519–525. https://doi.org/10.1016/S0925-3467(03)00087-9.

(6) Gourier, D.; Colle, L.; Lejus, A. M.; Vivien, D.; Moncorge, R. Electron-spin Resonance and Fluorescence Investigation of LaMgAl11O19:Ti3+, a Potential Tunable Laser Material. *J Appl Phys* **1998**, *63* (4), 1144. https://doi.org/10.1063/1.341139.

(7) Piotrowski, W.; Kuchowicz, M.; Dramićanin, M.; Marciniak, L. Lanthanide Dopant Stabilized $Ti^{3+}$ State and Supersensitive $Ti^{3+}$-Based Multiparametric Luminescent Thermometer in $SrTiO_3$:$Ln^{3+}$ ($Ln^{3+}$ = $Lu^{3+}$, $La^{3+}$, $Tb^{3+}$) Nanocrystals. *Chemical Engineering Journal* **2022**, *428*, 131165. https://doi.org/https://doi.org/10.1016/j.cej.2021.131165.

(8) Piotrowski, W. M.; Ristic, Z.; Dramićanin, M. D.; Marciniak, Ł. Modification of the Thermometric Performance of the Lifetime-Based Luminescent Thermometer Exploiting $Ti^{3+}$ Emission in $SrTiO_3$ and $CaTiO_3$ by Doping with Lanthanide Ions. *J Alloys Compd* **2022**, *906*, 164398. https://doi.org/10.1016/J.JALLCOM.2022.164398.

(9) Xiong, K.; Robertson, J.; Clark, S. J. Defect States in the High-Dielectric-Constant Gate Oxide LaAlO3. *Appl Phys Lett* **2006**, *89* (2). https://doi.org/10.1063/1.2221521.

(10) Gupta, M.; Rambadey, O. V.; Sagdeo, A.; Sagdeo, P. R. Investigating the Structural, Vibrational, Optical, and Dielectric Properties in Mg-Substituted LaAlO3. *Journal of Materials Science: Materials in Electronics* **2022**, *33* (16), 13352–13366. https://doi.org/10.1007/s10854-022-08273-y.

(11) Liu, Q.; Cheng, H.; Tu, T. Experiment and Simulation of Infrared Emissivity Properties of Doped LaAlO $_3$. *Journal of the American Ceramic Society* **2022**, *105* (4), 2713–2724. https://doi.org/10.1111/jace.18264.

(12) Yashaswini; Pratibha, S.; Lokesh, R.; Dhananjaya, N.; Pandurangappa, C. Disaccharide Assisted LaAlO3:Ce3+ Perovskite: Structural and Optical Studies Suitable for Display Devices. *Inorg Chem Commun* **2021**, *123*, 108342. https://doi.org/10.1016/j.inoche.2020.108342.

(13) Ye, J.; Bu, C.; Han, Z.; Wang, F.; Li, X.; Chen, Y.; Li, J. Flame-Spraying Synthesis and Infrared Emission Property of Ca2+/Cr3+ Doped LaAlO3 Microspheres. *J Eur Ceram Soc* **2015**, *35* (11), 3111–3118. https://doi.org/10.1016/j.jeurceramsoc.2015.04.039.

(14) Lim, S.-G.; Kriventsov, S.; Jackson, T. N.; Haeni, J. H.; Schlom, D. G.; Balbashov, A. M.; Uecker, R.; Reiche, P.; Freeouf, J. L.; Lucovsky, G. Dielectric Functions and Optical Bandgaps of High-K Dielectrics for Metal-Oxide-Semiconductor Field-Effect Transistors by Far Ultraviolet Spectroscopic Ellipsometry. *J Appl Phys* **2002**, *91* (7), 4500–4505. https://doi.org/10.1063/1.1456246.

(15) Edge, L. F.; Schlom, D. G.; Sivasubramani, P.; Wallace, R. M.; Holländer, B.; Schubert, J. Electrical Characterization of Amorphous Lanthanum Aluminate Thin Films Grown by Molecular-Beam Deposition on Silicon. *Appl Phys Lett* **2006**, *88* (11). https://doi.org/10.1063/1.2182019.

(16) Losurdo, M.; Giangregorio, M. M.; Luchena, M.; Capezzuto, P.; Bruno, G.; Toro, R. G.; Malandrino, G.; Fragalà, I. L.; Nigro, R. Lo. Structural–Optical Study of High-Dielectric-Constant

Oxide Films. *Appl Surf Sci* **2006**, *253* (1), 322–327. https://doi.org/10.1016/j.apsusc.2006.06.004.

(17) Cicerrella, E.; Freeouf, J. L.; Edge, L. F.; Schlom, D. G.; Heeg, T.; Schubert, J.; Chambers, S. A. Optical Properties of La-Based High-K Dielectric Films. *Journal of Vacuum Science & Technology A: Vacuum, Surfaces, and Films* **2005**, *23* (6), 1676–1680. https://doi.org/10.1116/1.2056555.

(18) Edge, L. F.; Schlom, D. G.; Chambers, S. A.; Cicerrella, E.; Freeouf, J. L.; Holländer, B.; Schubert, J. Measurement of the Band Offsets between Amorphous LaAlO3 and Silicon. *Appl Phys Lett* **2004**, *84* (5), 726–728. https://doi.org/10.1063/1.1644055.

(19) Mi, Y. Y.; Yu, Z.; Wang, S. J.; Lim, P. C.; Foo, Y. L.; Huan, A. C. H.; Ong, C. K. Epitaxial LaAlO3 Thin Film on Silicon: Structure and Electronic Properties. *Appl Phys Lett* **2007**, *90* (18). https://doi.org/10.1063/1.2736277.
